# Study on the attitude steering scheme of satellite platform for distributed spaceborne SAR

**DOI:** 10.1016/j.isci.2026.115314

**Published:** 2026-03-11

**Authors:** Haifeng Yu

**Affiliations:** 1 School of Energy and Power Engineering, Nanjing Institute of Technology, Nanjing 211167, China

**Keywords:** applied sciences, engineering

## Abstract

Compared with conventional synthetic aperture radar (SAR) on a single platform, distributed spaceborne SAR can realize imaging from multiple viewing angles by separating the transmitting and receiving satellite positions, so as to improve the capability of spaceborne SAR for a series of applications. To guarantee that echoes of distributed spaceborne SAR can be received completely and processed to generate images with high efficiency, we conduct deep research on the attitude steering scheme for the distributed SAR and build up the corresponding constraints on the transmitting and receiving satellite platforms from the perspectives of antenna beam footprint and bistatic Doppler centroid. Based on these constraints, four different attitude steering schemes have been brought forward in this paper, and their characteristics and performance are evaluated and compared via system simulations. In the end, the optimal approach for the distributed spaceborne SAR has been derived from the above analysis and simulations.

## Highlights

• Four different attitude steering schemes are proposed and designed for the distributed SAR
• Doppler frequency of received echoes corresponding to four schemes is analyzed and compared
• The overlapping ratio of transmitting and receiving beam footprints is analyzed and compared
• The optimal solution is derived from the virtual satellite and beam footprints overlapping

## Graphical abstract

**Figure:**
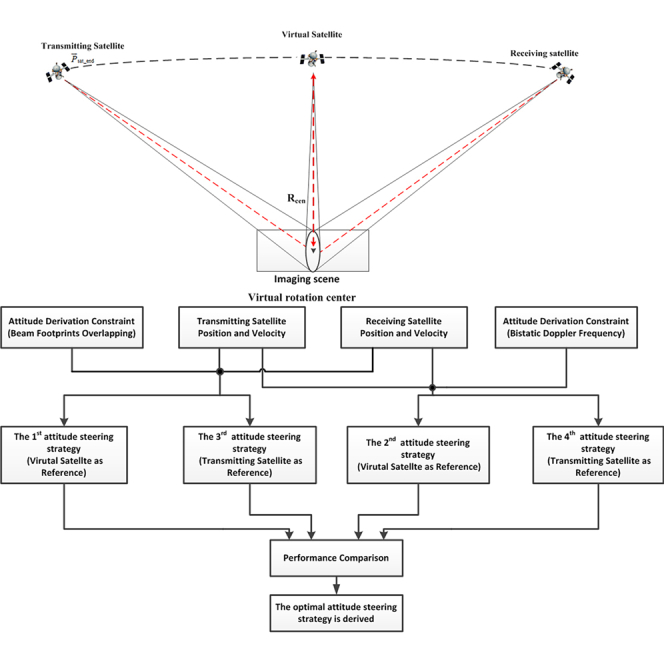

## Introduction

Distributed spaceborne synthetic aperture radar (SAR) missions open the door to a new dimension of radar remote sensing.^1^^,^^2^^,^^3^ One classic example is multibaseline cross-track interferometry for the generation of high-resolution digital elevation models (DEMs) with decimeter-level height accuracy. Future examples are multi-angular backscattering measurements for speckle reduction, improved scene characterization, and super-resolution. Multistatic SAR missions offer, moreover, promising perspectives for advanced vector deformation monitoring and 3-D velocity measurements of moving objects, the extraction of tropospheric water vapor and ionospheric delays in high turbulence, as well as the retrieval of ocean surface currents and topography together with their short- and long-term evolution over time. Taking the most prominent distributed spaceborne SAR-TanDEM-X as an example, its main goal is the generation of a global DEM.^4^ Besides that, new information products, such as DEM change maps (DCMs), have been developed. Beyond the operation products, a series of new science applications (such as urban morphology,^5^ forest maps^6^^,^^7^ and height measurement,^8^ lacier height measurement^9^ and mass balance evaluation,^10^ sea ice topography,^11^^,^^12^ lava field volume measurement,^13^ coastline detection and derivation^14^), along with the experimental data acquisitions (such as bistatic spaceborne SAR imaging,^15^ super resolution imaging,^16^ distributed SAR signal reconstruction,^17^^,^^18^ coherent azimuth ambiguities suppression,^19^^,^^20^ detection of maritime and land moving targets,^21^ SAR tomography for forest,^22^ bidirectional SAR imaging,^23^ Bistatic bidirectional SAR for ocean applications^24^), have been experimentally implemented with or are proposed for the TanDEM-X mission.

In addition to X-band close-formation SAR missions, the L-band, due to its penetration and high temporal coherence, is particularly suitable for deformation monitoring and has also received widespread attention. Currently, the only L-band distributed SAR formation is the Chinese LuTan-1 (LT-1). LT-1 operates at an orbit height of 607 km with a repeat cycle of eight days.^25^ It is an explicitly deformation monitoring-focused SAR constellation, which satisfies the requirements of disaster emergency response, land surveying, global forest resource surveying, biomass retrieval, and so forth. Additionally, it is able to achieve digital surface model (DSM) measurements with a grid resolution of 25 m^26^^,^^27^ in its close-formation phase.

Another typical but still in-development L-band close-formation distributed SAR is the TanDEM-L (TDL), which is a secondary spaceborne SAR identical to TerraSAR-L (TSL).^28^ It was proposed in 2009. The constellation orbits at a height of 745 km with a repeat cycle of 16 days. TDL offers various geophysical products related to the biosphere, hydrosphere, cryosphere, and lithosphere.^29^ In its close-formation stage, it can provide a DEM with a 12 m resolution. In 2017, TDL was in its final evaluation stage at the German Federal Ministry of Education and Research.^30^

In order to effectively reap the above potential benefits, the most important prerequisite is to guarantee the coordinated operations of transmitting (Tx) and receiving (Rx) satellite operations. This paper focuses on platform attitudes of both Tx and Rx satellites to realize this goal.

As regard to the imaging with conventional spaceborne SAR, attitude steering of satellite platform will be implemented on the yaw and pitch dimension to compensate the Doppler offset effects due to the Earth’s self-rotation, so as to make sure that Doppler centroid will be close to zero as much as possible along the range line of beam footprint to improve the imaging processing efficiency.^31^^,^^32^^,^^33^^,^^34^^,^^35^ For distributed spaceborne SAR, the bistatic Doppler centroid of received echoes will be jointly determined by positions, velocities, and attitudes of both transmitting and receiving satellites.^36^^,^^37^^,^^38^^,^^39^^,^^40^^,^^41^^,^^42^ Therefore, the attitude steering scheme should take into consideration the influences from both of them. Besides, to guarantee that two-way antenna gain and the consequent signal-to-noise ratio (SNR) meet their corresponding requirements, footprints of transmitting and receiving antenna beams should overlap with each other to the best.^43^^,^^44^ Thus, in order to achieve the above-mentioned goals, we bring forward specific and novel attitude steering schemes to deal with this issue.

In this paper, we select different satellites as reference (the first reference is the transmitting satellite, and the second one is the virtual satellite jointly determined by the transmitting and receiving satellites) and present a total of four different attitude steering schemes for distributed spaceborne SAR system, based on different constraints on the footprint overlapping or bistatic Doppler centroid variation along the range line. Furthermore, signal properties of received echoes corresponding to these four attitude steering schemes are analyzed and compared so as to derive the optimal option for different system operation scenarios.

### Yaw-pitch steering scheme for conventional spaceborne SAR

For conventional spaceborne SAR on a single platform, the Doppler centroid of received echoes *f*_*DC*_ can be expressed as

(Equation 1) $$f_{DC}=-\left(2/\lambda_{r}\right)\dot{\vec{r}}\cdot \hat{r}$$

where *λ*_*r*_ is the system wavelength, $\vec{r}$ is the space vector from the satellite position ${\vec{s}}_{P}$ to the ground target ${\vec{s}}_{T}$ and expressed as

(Equation 2) $$\vec{r}={\vec{s}}_{T}-{\vec{s}}_{P}$$

and $\hat{r}$ is the unit vector of $\vec{r}$, and given by

(Equation 3) $$\hat{r}=\vec{r}/\left\|\vec{r}\right\|$$

From Equation 1, it can be seen that in order to realize the goal of *f*_*DC*_ = 0, the corresponding requirement that should be met is

(Equation 4) $$\dot{\vec{r}}\cdot \vec{r}=\left({\dot{\vec{s}}}_{T}-{\dot{\vec{s}}}_{P}\right)\cdot \left({\vec{s}}_{T}-{\vec{s}}_{P}\right)=0$$

where ${\dot{\vec{s}}}_{T}$ is the target velocity attributed to the Earth’s self-rotation and thus can be expressed as

(Equation 5) $${\dot{\vec{s}}}_{T}={\vec{\omega }}_{E}\times {\vec{s}}_{T}$$

Then, the above requirement can be rewritten as

(Equation 6) $$\left({\vec{\omega }}_{E}\times {\vec{s}}_{T}-{\dot{\vec{s}}}_{P}\right)\cdot \left({\vec{s}}_{T}-{\vec{s}}_{P}\right)=\left({\vec{\omega }}_{E}\times {\vec{s}}_{T}\right)\cdot {\vec{s}}_{T}-\left({\vec{\omega }}_{E}\times {\vec{s}}_{T}\right)\cdot {\vec{s}}_{P}-{\dot{\vec{s}}}_{P}\left({\vec{s}}_{T}-{\vec{s}}_{P}\right)=0$$

Based on the characteristics of the cross product, Equation 6 can be further rewritten as

(Equation 7) $$-\left({\vec{s}}_{P}\times {\vec{\omega }}_{E}\right)\cdot {\vec{s}}_{T}-{\dot{\vec{s}}}_{P}\cdot \left({\vec{s}}_{T}-{\vec{s}}_{P}\right)=0$$

Replacing the term ${\vec{s}}_{T}$ by ${\vec{s}}_{P}+\vec{r}$ and $\left({\vec{s}}_{T}-{\vec{s}}_{P}\right)$ by $\vec{r}$ in the above equation, we have

(Equation 8) $$-\left({\vec{s}}_{P}\times {\vec{\omega }}_{E}\right)\cdot \left({\vec{s}}_{P}+\vec{r}\right)-{\dot{\vec{s}}}_{P}\cdot \vec{r}=\left({\dot{\vec{s}}}_{P}-{\vec{\omega }}_{E}\times {\vec{s}}_{P}\right)\cdot \vec{r}=0$$

To facilitate the following analysis and derivation, we define a new variable—the relative satellite velocity ${\vec{v}}_{\text{rel}}={\dot{\vec{s}}}_{P}-{\vec{\omega }}_{E}\times {\vec{s}}_{P}$. From Equation 8, we can see that in order to guarantee the Doppler centroid *f*_*DC*_ = 0, the antenna beam center vector should be normal to the ${\vec{v}}_{\text{rel}}$. Therefore, the attitude adjustment should be implemented in the yaw and pitch dimensions to make sure that the X axis of the antenna beam coincides with the ${\vec{v}}_{\text{rel}}$, the Y-Z plane of the antenna beam, as well as the beam center within this plane, will always be normal to the velocity vector ${\vec{v}}_{\text{rel}}$ irrespective of the looking angle of the beam center.

**Figure 1:**
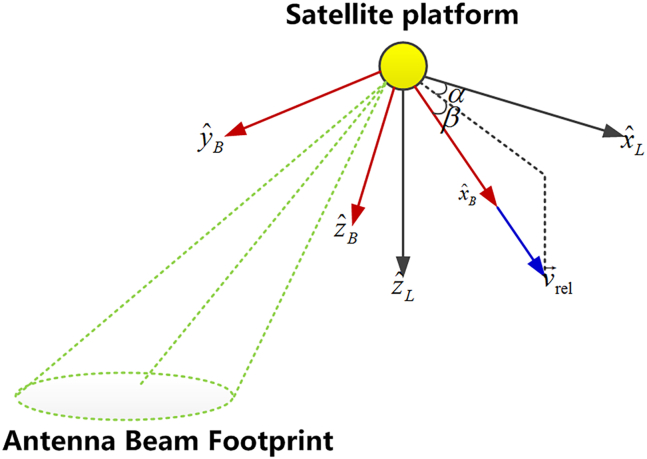
The yaw-pitch attitude steering process of conventional spaceborne SAR

In the local vertical, local horizontal (LVLH) coordinate system, the analytical expression for ${\vec{v}}_{\text{rel}}$ is given by

(Equation 9) $${\vec{v}}_{\text{rel}}=v_{r,x}^{L}{\hat{x}}_{L}+v_{r,y}^{L}{\hat{y}}_{L}+v_{r,z}^{L}{\hat{z}}_{L}$$

where $v_{r,x}^{L}$, $v_{r,y}^{L}$, and $v_{r,z}^{L}$ are three-axis components of ${\vec{v}}_{\text{rel}}$, ${\hat{x}}_{L}$, ${\hat{y}}_{L}$, and ${\hat{z}}_{L}$ are unit vectors of the three axes of the coordinate system. If the platform did not perform attitude steering, the three axes of the satellite body would exactly coincide with ${\hat{x}}_{L}$, ${\hat{y}}_{L}$ and ${\hat{z}}_{L}$. Then, in the yaw-pitch-roll (YPR) rotation sequence, the corresponding yaw-steering angle *θ*_yaw_ and pitch-steering angle *θ*_pitch_ are

(Equation 10) $$\theta_{\text{yaw}}=\tan^{-1}\left(v_{r,y}^{L}/v_{r,x}^{L}\right)$$

and

(Equation 11) $$\theta_{\text{pitch}}=-\tan^{-1}\left(v_{r,z}^{L}/\sqrt{{\left(v_{r,x}^{L}\right)}^{2}+{\left(v_{r,y}^{L}\right)}^{2}}\right)$$

respectively, to realize the complete superposition of th X axis of the satellite with the relative velocity ${\vec{v}}_{rel}$. This attitude steering process is illustrated in Figure 1.

### Attitude steering strategy for master and slave satellites of distributed spaceborne SAR system

The range history of echoes for distributed spaceborne SAR can be modeled as “transmitting satellite – observation scene – receiving satellite.” Thus, the derivation of bistatic Doppler frequency *f*_BiD_ should take into consideration both the transmitting and receiving satellites, and it can be expressed as

(Equation 12) $$f_{\text{BiD}}=-\left({\vec{r}}_{\text{Tx}}\cdot {\hat{r}}_{\text{Tx}}\right)/\lambda_{r}-\left({\vec{r}}_{\text{Rx}}\cdot {\hat{r}}_{\text{Rx}}\right)/\lambda_{r}=f_{DTx}+f_{DRx}$$

where ${\vec{r}}_{\text{Tx}}$ is the vector from the transmitting satellite to the target in the scene given by ${\vec{r}}_{\text{Tx}}={\vec{s}}_{T}-{\vec{s}}_{\text{Tx}}$ and ${\hat{r}}_{\text{Tx}}$ is its unit vector, ${\vec{r}}_{\text{Rx}}$ is the vector from the receiving satellite to the target given, and ${\hat{r}}_{\text{Rx}}$ is its unit vector.

**Figure 2:**
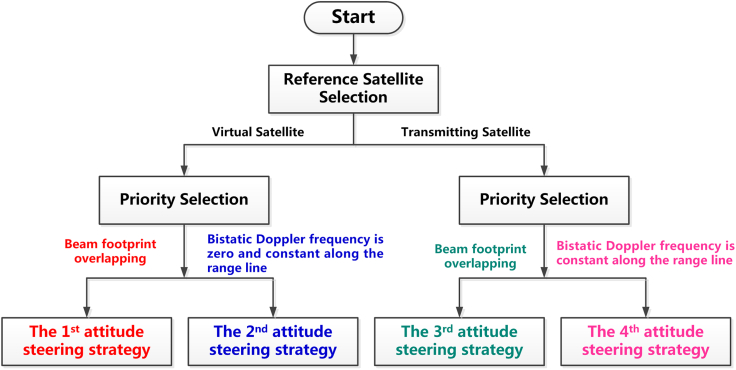
The block diagram of a mind map for four different bistatic attitude steering strategies

It can be seen from Equation 12 that the Doppler frequency of distributed spaceborne SAR is jointly determined by two independent terms. The first one is transmitting Doppler frequency *f*_*DTx,*_ and the second is the receiving Doppler frequency *f*_*DRx*_. Hence, we can perform the attitude steering on either the transmitting satellite or the receiving satellite or both of them to achieve the goal of a constant bistatic Doppler frequency along the range line. In the following, we will present four different attitude steering strategies based on the selection of the reference satellite and the corresponding requirement on either beam footprint overlapping or bistatic Doppler frequency. The difference among these four attitude steering strategies is illustrated by Figure 2. By selecting different reference satellites (virtual satellite or transmitting satellite) and the design priority (footprint overlapping or bistatic Doppler frequency constant) of attitude, these four strategies can be distinguished from each other clearly.

### Attitude steering based on a virtual satellite and footprint overlapping

**Figure 3:**
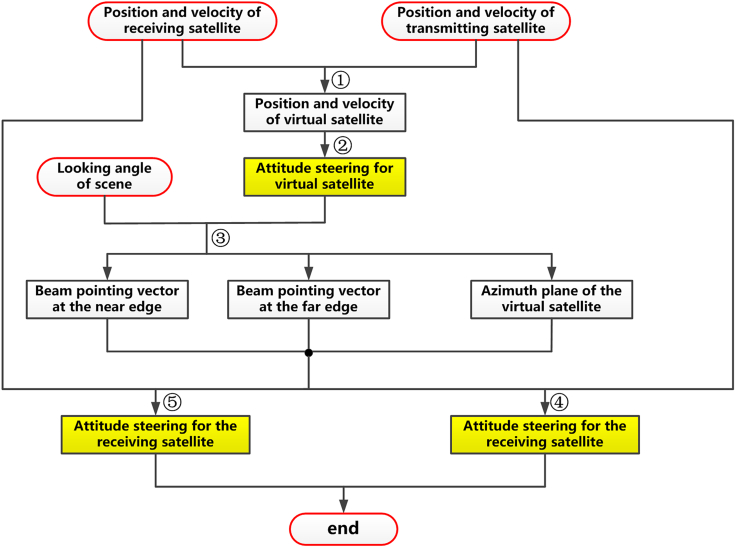
The flowchart of the 1^st^ attitude steering strategy for distributed spaceborne SAR

In the first attitude steering strategy, we select the virtual satellite, whose position and velocity vectors are jointly determined by their counterparts of transmitting and receiving satellites, as the reference satellite. The constraint for this strategy is that it should realize the overlapping of antenna beam footprints of transmitting and receiving satellites as much as possible. The flowchart for this strategy is illustrated in Figure 3, and it consists of 5 steps in the whole process. In the following, we will give the details of these 5 steps.

Step 1: Determination of the position and velocity of the virtual satellite

Based on the position and velocity knowledge of both transmitting and receiving satellites, we could determine the virtual satellite, whose position and velocity are given by

(Equation 13) $${\vec{s}}_{\text{vx}}=\left({\vec{s}}_{\text{Tx}}+{\vec{s}}_{\text{Rx}}\right)/2$$

and

(Equation 14) $${\vec{v}}_{\text{vx}}=\left({\vec{v}}_{\text{Tx}}+{\vec{v}}_{\text{Rx}}\right)/2$$

respectively. It can be seen that they are the exact arithmetic mean values of their respective counterparts of transmitting and receiving satellites.

Step 2: Determination of the attitude steering strategy of the virtual satellite

With the position and velocity information obtained by (Equation 13), (Equation 14), we could acquire it attitude steering angles in the yaw and pitch dimensions using (Equation 9), (Equation 10), (Equation 11). Assuming the looking angle of the observation scene center is *α*_*c*_, the roll angle is then directly acquired as

(Equation 15) $$\theta_{\text{roll}}=\alpha_{c}$$

in the YPR rotation sequence.

Step 3: Determining the vector from the virtual satellite to the near and far edge

With the above Euler angles of the virtual satellite, we could determine the unit vectors of its three axes as

(Equation 16) $$\left[{\hat{x}}_{v},{\hat{y}}_{v},{\hat{z}}_{v}\right]=M_{\text{orb}\to \text{ECEF}}\cdot M_{\text{roll}}\cdot M_{\text{pitch}}\cdot M_{\text{yaw}}$$

where ${\hat{x}}_{v}$, ${\hat{y}}_{v}$, and ${\hat{z}}_{v}$ are the unit vectors corresponding to X-, Y-, and z axis respectively, and *M*_yaw_, *M*_pitch_, and *M*_roll_ are the rotation matrices of yaw, pitch, and roll angles, respectively. Then, we can further determine vectors from the virtual satellite to the near and far limits of the scene, and as

(Equation 17) $${\vec{r}}_{n}=-{\hat{y}}_{v}\cdot \sin\left(\theta_{n}\right)+{\hat{z}}_{v}\cdot \cos\left(\theta_{n}\right)$$

and

(Equation 18) $${\vec{r}}_{f}=-{\hat{y}}_{v}\cdot \sin\left(\theta_{f}\right)+{\hat{z}}_{v}\cdot \cos\left(\theta_{f}\right)$$

respectively. *θ*_n_ and *θ*_f_ are offset angles with respect to the looking angle of the scene center associated with the near and far edges of the scene.

Step 4: Determination of the attitude steering strategy of the transmitting satellite

**Figure 4:**
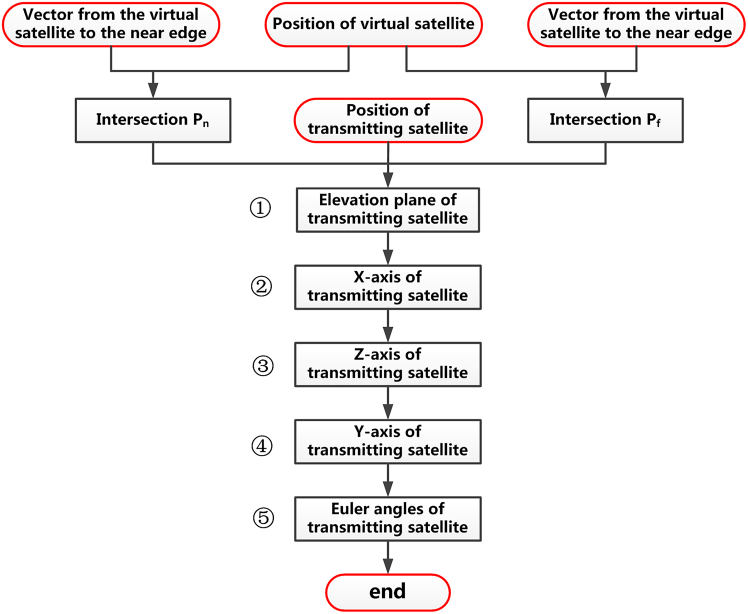
The flowchart of the attitude steering strategy for the transmitting satellite

Up to now, the position of the virtual satellite and vectors to the near and far edges of the scene from this position have all been determined. We can then derive the corresponding attitude steering strategy for the transmitting and receiving satellite. Without loss of generality, we select the transmitting satellite as an example to illustrate this procedure, whose flowchart is given by Figure 4. It can be seen that this step can be further subdivided into 5 steps, which are.

• Determination of the elevation plane of the transmitting satellite;
• Determination of the X axis unit vector of the transmitting satellite;
• Determination of the z axis unit vector of the transmitting satellite;
• Determination of the Y axis unit vector of the transmitting satellite;
• Determination of Euler angles of transmitting satellite.
Step 4.1: Determination of the elevation plane of the transmitting satellite

With the position of a virtual satellite ${\vec{s}}_{\text{vx}}$ and vectors ${\hat{r}}_{n}$ and ${\hat{r}}_{f}$, we can obtain the intersections of these two vectors with the Earth’s surface ${\vec{p}}_{n}$ and ${\vec{p}}_{f}$, respectively. Then, vectors from the transmitting satellite to them are given by

(Equation 19) $${\vec{r}}_{\text{an}}={\vec{p}}_{n}-{\vec{s}}_{\text{Tx}}$$

and

(Equation 20) $${\vec{r}}_{\text{af}}={\vec{p}}_{f}-{\vec{s}}_{\text{Tx}}$$

respectively. Since these two vectors, as well as the transmitting satellite position ${\vec{s}}_{\text{Tx}}$, all lie within the elevation plane Δ*S*_el_, this plane can be defined by the following two constraints

(Equation 21) $$\left\{ \begin{array}{l} {\vec{s}}_{\text{Tx}}\in \Delta S_{\text{el}}\\ \left({\vec{r}}_{\text{an}}\times {\vec{r}}_{\text{af}}\right)⊥\Delta S_{\text{el}} \end{array}\right.$$

of which the first one indicates that ${\vec{s}}_{\text{Tx}}$ is within the plane, and the second implies that Δ*S*_el_ should be normal to the cross product of $\left({\vec{r}}_{\text{an}}\times {\vec{r}}_{\text{af}}\right)$.

Step 4.2: Determination of the X axis unit vector of the transmitting satellite

The X axis vector ${\hat{x}}_{\text{Tx}}$ should be normal to the elevation plane Δ*S*_el_. Therefore, it can be determined by the cross product of ${\vec{r}}_{\text{an}}$ and ${\vec{r}}_{\text{af}}$. When the satellite operates in the left-side looking geometry, ${\hat{x}}_{\text{Tx}}$ can be expressed as

(Equation 22) $${\hat{x}}_{\text{Tx}}=\left({\vec{r}}_{\text{an}}\times {\vec{r}}_{\text{af}}\right)/\left\|{\vec{r}}_{\text{an}}\times {\vec{r}}_{\text{af}}\right\|$$

where ‖·‖ is the Euclidean norm operation; otherwise, it is given by

(Equation 23) $${\hat{x}}_{\text{Tx}}=\left({\vec{r}}_{\text{af}}\times {\vec{r}}_{\text{an}}\right)/\left\|{\vec{r}}_{\text{af}}\times {\vec{r}}_{\text{an}}\right\|$$

if the scene is on the right side of the satellite.

Step 4.3: Determination of z axis unit vector of the transmitting satellite

To guarantee that the antenna beam footprint can effectively cover the swath, the intersection of its z axis vector ${\hat{z}}_{\text{Tx}}$ with the Earth’s surface should be located with the azimuth plane of the virtual satellite Δ*M*_az_. Assuming the coordinates of this intersection ${\vec{p}}_{\text{az}}$ are (*x*_az_,y_az_,*z*_az_), they can thus be derived from the following three equations

(Equation 24) $$\left({\vec{p}}_{\text{az}}-{\vec{s}}_{\text{Tx}}\right)\cdot {\vec{x}}_{\text{Tx}}=0$$

(Equation 25) $$\left({\vec{p}}_{\text{az}}-{\vec{s}}_{\text{vx}}\right)\cdot {\vec{y}}_{v}=0$$

(Equation 26) $$x_{\text{az}}^{2}/R_{a}^{2}+y_{\text{az}}^{2}/R_{a}^{2}+z_{\text{az}}^{2}/R_{b}^{2}=1$$

where *R*_a_ and *R*_b_ are the equatorial radius and polar radius of the Earth, respectively. Once the ${\vec{p}}_{\text{az}}$ is obtained, the unit vector of the z axis is given by

(Equation 27) $${\vec{z}}_{\text{Tx}}=\left({\vec{p}}_{\text{az}}-{\vec{s}}_{\text{Tx}}\right)/\left\|{\vec{p}}_{\text{az}}-{\vec{s}}_{\text{Tx}}\right\|$$

Step 4.4: Determination of the Y axis unit vector of the transmitting satellite

Based on the X axis vector and the z axis vector of the transmitting satellite, its Y axis vector ${\vec{y}}_{\text{Tx}}$ can then be derived with the theorem of the right-hand rule and given by

(Equation 28) $${\hat{y}}_{\text{Tx}}={\hat{z}}_{\text{Tx}}\times {\hat{x}}_{\text{Tx}}$$

Step 4.5: Determination of Euler angles of transmitting satellite

Based on the above ${\hat{x}}_{\text{Tx}}$, ${\hat{y}}_{\text{Tx}}$ and ${\hat{z}}_{\text{Tx}}$, along with the position and velocity of the transmitting satellite ${\vec{s}}_{\text{Tx}}$ and ${\vec{v}}_{\text{Tx}}$, its Euler angles in the yaw, pitch, and roll dimensions $\theta_{\text{yaw}\_\text{Tx}}$, $\theta_{\text{pitch}\_\text{Tx}}$ and $\theta_{\text{roll}\_\text{Tx}}$ can then be obtained consequently.

Step 5: Determination of the attitude steering strategy of the receiving satellite

**Figure 5:**
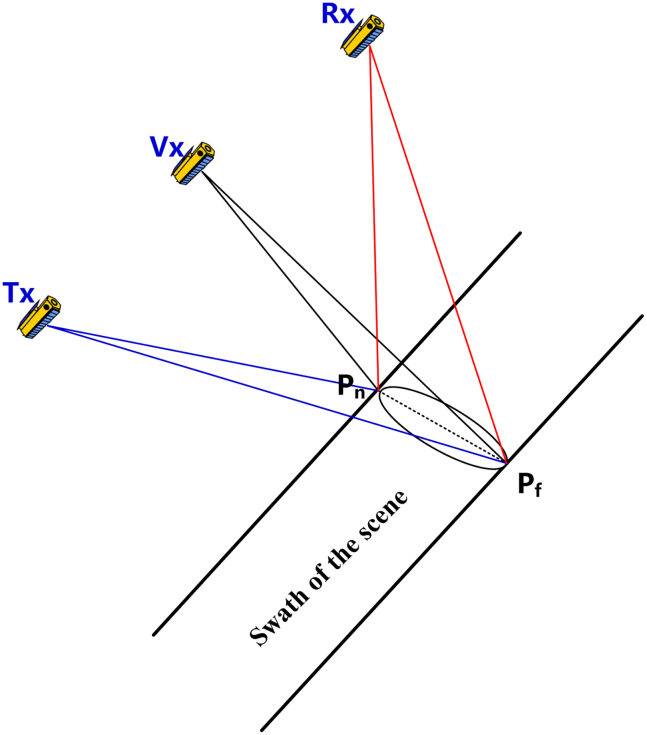
The illustration of the implementation of the 1^st^ attitude steering strategy

The attitude steering strategy of a receiving satellite can be determined in a similar way to that of a transmitting satellite. Utilizing the position and velocity of the receiving satellite ${\vec{s}}_{\text{Rx}}$ and ${\vec{v}}_{\text{Rx}}$, along with procedures presented in Step 4.1–4.5, one can obtain the unit vector of the three axes of the receiving satellite ${\hat{x}}_{\text{Rx}}$, ${\hat{y}}_{\text{Rx}}$ and ${\hat{z}}_{\text{Rx}}$. Finally, three Euler angles $\theta_{\text{yaw}\_\text{Rx}}$, $\theta_{\text{pitch}\_\text{Rx}}$ and $\theta_{\text{roll}\_\text{Rx}}$ can be determined. This implementation of the strategy can be briefly illustrated by Figure 5, and one can observe that it can realize the overlapping of beam footprints to the best.

### Attitude steering based on virtual satellite and bistatic Doppler centroid

**Figure 6:**
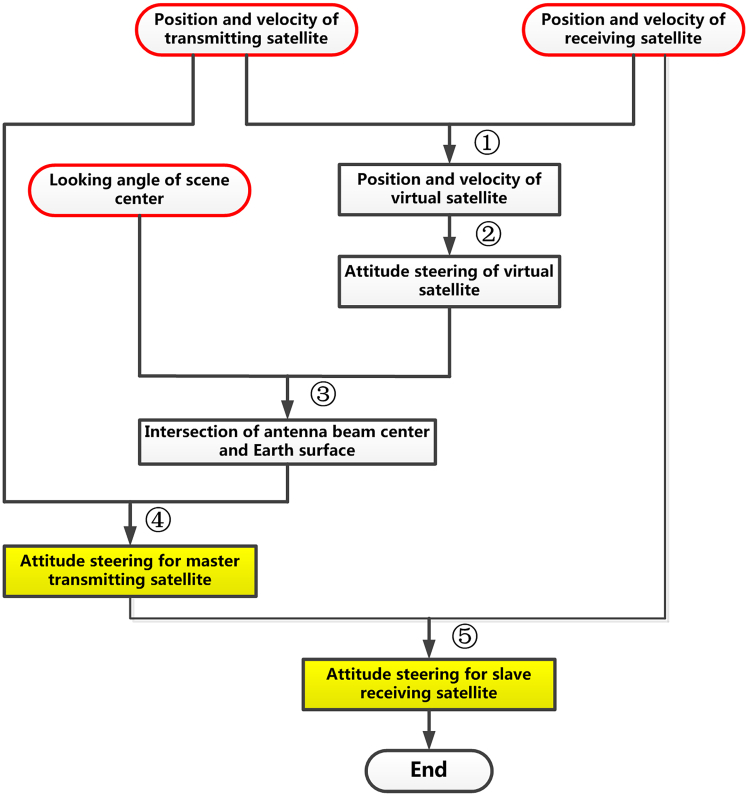
The flowchart of the 2^nd^ attitude steering strategy for distributed spaceborne SAR

The constraint of this attitude steering strategy requires that the bistatic Doppler centroid *f*_Bid_ = 0 along the range dimension rather than the complete footprints overlapping. Therefore, the attitude steering approaches of both the transmitting satellite and the receiving satellite in this subsection are quite different from their counterparts in the last subsection. Figure 6 shows the general flowchart of this type of attitude steering strategy, which consists of 5 steps. In the following, we will present a detailed description of each step.

Step 1: Determination of the position and velocity of the virtual satellite

Once positions and velocities of the transmitting and receiving satellites have been given, the position and velocity of the virtual satellite can be determined in the same way as that in the 1^st^ attitude strategy, which are expressed in (Equation 13), (Equation 14), respectively.

Step 2: Determine the attitude steering strategy of the virtual satellite

This step is identical to that in the last subsection. The roll angle of the virtual satellite $\theta_{\text{roll}\_\text{vx}}$ is directly determined by the looking angle of the scene center *θ*_look_ as

(Equation 29) $$\theta_{\text{roll}\_\text{vx}}=\theta_{\text{look}}$$

Step 3: Determine the intersection of the virtual antenna beam center and the Earth’s surface

With the Euler angles of the virtual satellite obtained above, we can further determine the unit vectors of the three axes ${\hat{x}}_{v}$, ${\hat{y}}_{v}$ and ${\hat{z}}_{v}$. Then, the intersection of the virtual antenna beam center and the Earth’s surface ${\vec{p}}_{\text{mc}}$ can be determined consequently. This point position will be used to determine the attitude steering of the transmitting satellite.

Step 4: Determine the attitude steering strategy of the transmitting satellite

In this subsection, the attitude steering of the transmitting satellite will be defined by its position and velocity, along with the point ${\vec{p}}_{\text{mc}}$. The corresponding constraint for its steering is that the transmitting Doppler frequency *f*_*DTx*_ will be kept constant along the range line of its antenna beam footprint.

Based on the position of ${\vec{p}}_{\text{mc}}$ and the transmitting satellite position ${\vec{s}}_{\text{Tx}}$, the vector of its z axis ${\vec{z}}_{\text{Tx}}$ can be determined by

(Equation 30) $${\vec{z}}_{\text{Tx}}={\vec{p}}_{\text{mc}}-{\vec{s}}_{\text{Tx}}$$

and the corresponding unit vector of the z axis ${\hat{z}}_{\text{Tx}}$ is given by

(Equation 31) $${\hat{z}}_{\text{Tx}}={\vec{z}}_{\text{Tx}}/\left\|{\vec{z}}_{\text{Tx}}\right\|$$

To design the Y axis vector of the transmitting satellite ${\vec{y}}_{\text{Tx}}$, it should be kept in mind that the Doppler frequency *f*_*DTx*_ will not vary with the nadir looking angle. As a consequence, ${\vec{y}}_{\text{Tx}}$ can be determined as the cross product of ${\vec{z}}_{\text{Tx}}$ and the velocity of the transmitting platform ${\vec{v}}_{\text{Tx}}$

(Equation 32) $${\vec{y}}_{\text{Tx}}={\vec{z}}_{\text{Tx}}\times {\vec{v}}_{\text{Tx}}$$

In this way, the unit vector of the Y axis is given by

(Equation 33) $${\hat{y}}_{\text{Tx}}={\vec{y}}_{\text{Tx}}/\left\|{\vec{y}}_{\text{Tx}}\right\|$$

At last, the X axis vector of the transmitting satellite ${\hat{x}}_{\text{Tx}}$ can be jointly defined by ${\hat{z}}_{\text{Tx}}$ and ${\hat{y}}_{\text{Tx}}$ based on the theorem of the right-hand rule as

(Equation 34) $${\hat{x}}_{\text{Tx}}={\hat{y}}_{\text{Tx}}\times {\hat{z}}_{\text{Tx}}$$

Based on the unit vectors of three axes given by (Equation 31), (Equation 33), (Equation 34), the Euler angles of yaw, pitch, and roll corresponding to the transmitting satellite can be derived.

Step 5: Determine the attitude steering strategy of the receiving satellite

With regard to the attitude steering strategy of receiving satellite, its corresponding requirements are that the bistatic Doppler frequency should be zero at the near and far ends of the receiving antenna beams, as defined by the following equations.

(Equation 35) $$f_{\text{bi}\_\text{near}}=0$$

and

(Equation 36) $$f_{\text{bi}\_\text{far}}=0$$

**Figure 7:**
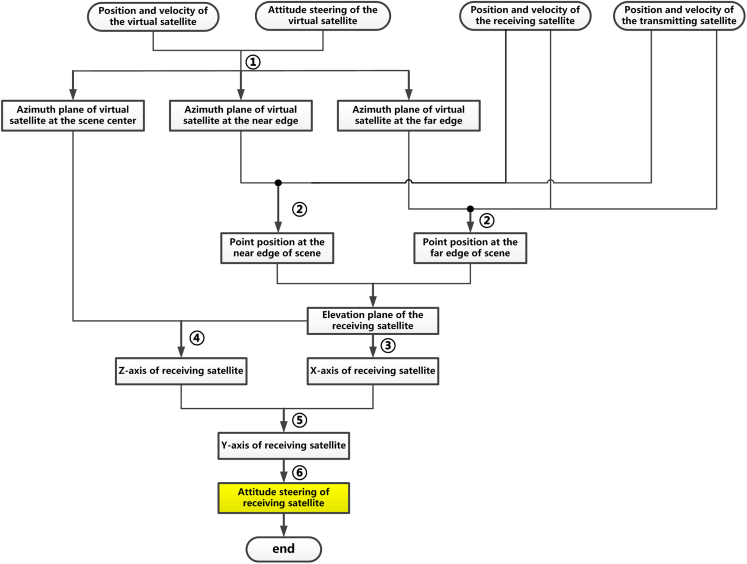
The flowchart of the attitude steering strategy for the receiving satellite

The detailed procedures of determining the attitude steering strategy of receiving satellite are presented in Figure 7, and one can see that it is comprised of 6 steps that are.

• Determination of azimuth planes of virtual satellite at the near, central, and far edges of the scene;
• Searching out the position within these azimuth planes of a virtual satellite meeting the requirement of *f*_bistatic_ = 0;
• Determination of the X axis vector of the receiving satellite;
• Determination of the z axis vector of the receiving satellite;
• Determination of the Y axis vector of the receiving satellite;
• Determination of attitude steering strategy for the receiving satellite
Step 5.1: Determination of azimuth planes of virtual satellite at the near, central, and far edge of swath

With the unit vectors of the three axes of the virtual satellite ${\hat{x}}_{v}$, ${\hat{y}}_{v}$, and ${\hat{z}}_{v}$, along with its position ${\vec{s}}_{\text{vx}}$ and ${\vec{v}}_{\text{vx}}$, the azimuth plane of the virtual satellite at the center of the scene $\Delta S_{\text{az}\_\text{cen}}$ can be determined by the following constraints

(Equation 37) $$\left\{ \begin{array}{l} {\vec{s}}_{\text{vx}}\in \Delta S_{\text{az}\_\text{cen}}\\ {\hat{y}}_{v}⊥\Delta S_{\text{az}\_\text{cen}} \end{array}\right.$$

Assuming the looking angle width of the scene is *θ*_s_, the normal vectors of the azimuth plane at the near and far edges of the scene are given by

(Equation 38) $${\hat{y}}_{v\_\text{near}}={\hat{y}}_{v}\;\cos\left(\theta_{s}/2\right)+{\hat{z}}_{v}\;\cos\left(\theta_{s}/2\right)$$

and

(Equation 39) $${\hat{y}}_{v\_\text{far}}={\hat{y}}_{v}\;\cos\left(\theta_{s}/2\right)-{\hat{z}}_{v}\;\cos\left(\theta_{s}/2\right)$$

respectively. Then, the azimuth planes of the virtual satellite at the near and far edges of the swath can be defined in the same way asin Equation 37, and they are given by

(Equation 40) $$\left\{ \begin{array}{l} {\vec{s}}_{\text{vx}}\in \Delta S_{\text{az}\_\text{near}}\\ {\hat{y}}_{v\_\text{near}}⊥\Delta S_{\text{az}\_\text{near}} \end{array}\right.$$

and

(Equation 41) $$\left\{ \begin{array}{l} {\vec{s}}_{\text{vx}}\in \Delta S_{\text{az}\_\text{far}}\\ {\hat{y}}_{v\_\text{far}}⊥\Delta S_{\text{az}\_\text{far}} \end{array}\right.$$

respectively.

After obtaining these two azimuth planes, we will search out the position within each of them to satisfy the constraints shown in (Equation 35), (Equation 36).

Step 5.2: Searching out the positions that meet the requirement *f*_bistatic_ = 0

In this subsection, we adopt the azimuth plane at the near edge as an example to illustrate how to obtain the required position ${\vec{p}}_{n}$. Since this position is also located on the surface of the Earth, the corresponding equations for its derivation can be listed as follows.

(Equation 42) $$\left({\vec{p}}_{n}-{\vec{s}}_{\text{vx}}\right)\cdot {\hat{y}}_{v\_\text{near}}=0$$

(Equation 43) $${\left[{\vec{p}}_{n}\left(x\right)\right]}^{2}/R_{a}^{2}+{\left[{\vec{p}}_{n}\left(y\right)\right]}^{2}/R_{a}^{2}+{\left[{\vec{p}}_{n}\left(z\right)\right]}^{2}/R_{b}^{2}=1$$

(Equation 44) $$-\frac{\left({\vec{p}}_{n}-{\vec{s}}_{\text{Tx}}\right)}{\lambda \left\|{\vec{p}}_{n}-{\vec{s}}_{\text{Tx}}\right\|}\cdot {\vec{v}}_{\text{Tx}}-\frac{\left({\vec{p}}_{n}-{\vec{s}}_{\text{Rx}}\right)}{\lambda \left\|{\vec{p}}_{n}-{\vec{s}}_{\text{Rx}}\right\|}\cdot {\vec{v}}_{\text{Rx}}=0$$

The first equation indicates that the vector from the virtual satellite position ${\vec{s}}_{\text{vx}}$ should be normal to the vector ${\hat{y}}_{v\_\text{near}}$. The second one implies the point ${\vec{p}}_{n}$ lies on the surface of the earth, and the third one is the exact requirement for $f_{\text{bi}\_\text{near}}=0$.

Similarly, the point position in the azimuth plane of the far edge ${\vec{p}}_{f}$ can also be found out. And the corresponding requirements are modified as

(Equation 45) $$\left({\vec{p}}_{f}-{\vec{s}}_{\text{vx}}\right)\cdot {\hat{y}}_{v\_\text{far}}=0$$

(Equation 46) $${\left[{\vec{p}}_{f}\left(x\right)\right]}^{2}/R_{a}^{2}+{\left[{\vec{p}}_{f}\left(y\right)\right]}^{2}/R_{a}^{2}+{\left[{\vec{p}}_{f}\left(z\right)\right]}^{2}/R_{b}^{2}=1$$

(Equation 47) $$-\frac{\left({\vec{p}}_{f}-{\vec{s}}_{\text{Tx}}\right)}{\lambda \left\|{\vec{p}}_{f}-{\vec{s}}_{\text{Tx}}\right\|}\cdot {\vec{v}}_{\text{Tx}}-\frac{\left({\vec{p}}_{f}-{\vec{s}}_{\text{Rx}}\right)}{\lambda \left\|{\vec{p}}_{f}-{\vec{s}}_{\text{Rx}}\right\|}\cdot {\vec{v}}_{\text{Rx}}=0$$

With the positions of these two specific points, we can then determine the unit vectors of the three axes of the receiving satellite.

Step 5.3: Determination of X axis unit vector of receiving satellite

The elevation plane of the receiving satellite should contain ${\vec{p}}_{n}$ and ${\vec{p}}_{f}$ acquired in the last subsection to make sure the bistatic Doppler frequency along the range line of the antenna beam footprint is equal to zero. Thus, both of these vectors $\left({\vec{p}}_{n}-{\vec{s}}_{\text{Rx}}\right)$ and $\left({\vec{p}}_{f}-{\vec{s}}_{\text{Rx}}\right)$ should lie within the elevation plane. Since the X axis vector of the receiving satellite is normal to its elevation plane, it can be determined by the cross product of $\left({\vec{p}}_{n}-{\vec{s}}_{\text{Rx}}\right)$ and $\left({\vec{p}}_{s}-{\vec{s}}_{\text{Rx}}\right)$ as

(Equation 48) $${\vec{x}}_{\text{Rx}}=\left({\vec{p}}_{f}-{\vec{s}}_{\text{Rx}}\right)\times \left({\vec{p}}_{n}-{\vec{s}}_{\text{Rx}}\right)$$

Here, we suppose the satellite operates in the right-side looking geometry; otherwise, it is given by

(Equation 49) $${\vec{x}}_{\text{Rx}}=\left({\vec{p}}_{n}-{\vec{s}}_{\text{Rx}}\right)\times \left({\vec{p}}_{f}-{\vec{s}}_{\text{Rx}}\right)$$

Consequently, its unit vector can be expressed as

(Equation 50) $${\hat{x}}_{\text{Rx}}={\vec{x}}_{\text{Rx}}/\left\|{\vec{x}}_{\text{Rx}}\right\|$$

Step 5.4: Determination of the z axis unit vector of the receiving satellite

Once the elevation plane has been determined, the z axis vector should lie within it. Besides, to make sure that the beam footprint of the receiving satellite can overlap with that of the virtual as well as that of the transmitting satellite, the intersection of the z axis vector and the Earth’s surface should be located within the azimuth plane of the virtual satellite at the center of the scene. Therefore, to determine the z axis vector of the receiving satellite, its intersection with the Earth’s surface ${\vec{p}}_{c}$ should be derived first. Based on the above analysis, its corresponding constraints can be expressed as the following three equations

(Equation 51) $$\left({\vec{p}}_{c}-{\vec{s}}_{\text{Rx}}\right)\cdot {\hat{x}}_{\text{Rx}}=0$$

(Equation 52) $$\left({\vec{p}}_{c}-{\vec{s}}_{\text{vx}}\right)\cdot {\hat{y}}_{v}=0$$

(Equation 53) $${\left[{\vec{p}}_{c}\left(x\right)\right]}^{2}/R_{a}^{2}+{\left[{\vec{p}}_{c}\left(y\right)\right]}^{2}/R_{a}^{2}+{\left[{\vec{p}}_{c}\left(z\right)\right]}^{2}/R_{b}^{2}=1$$

Then, the z axis vector of the receiving satellite ${\vec{z}}_{\text{Rx}}$ can be expressed as

(Equation 54) $${\vec{z}}_{\text{Rx}}={\vec{p}}_{c}-{\vec{s}}_{\text{Rx}}$$

and its unit vector ${\hat{z}}_{\text{Rx}}$ is given by

(Equation 55) $${\hat{z}}_{\text{Rx}}={\vec{z}}_{\text{Rx}}/\left\|{\vec{z}}_{\text{Rx}}\right\|$$

Step 5.5: Determination of the Y axis unit vector of the receiving satellite

With the X axis and z axis unit vectors of the receiving satellite, the Y axis vector can be derived using the right-hand rule as

(Equation 56) $${\hat{y}}_{\text{Rx}}={\hat{z}}_{\text{Rx}}\times {\hat{x}}_{\text{Rx}}$$

**Figure 8:**
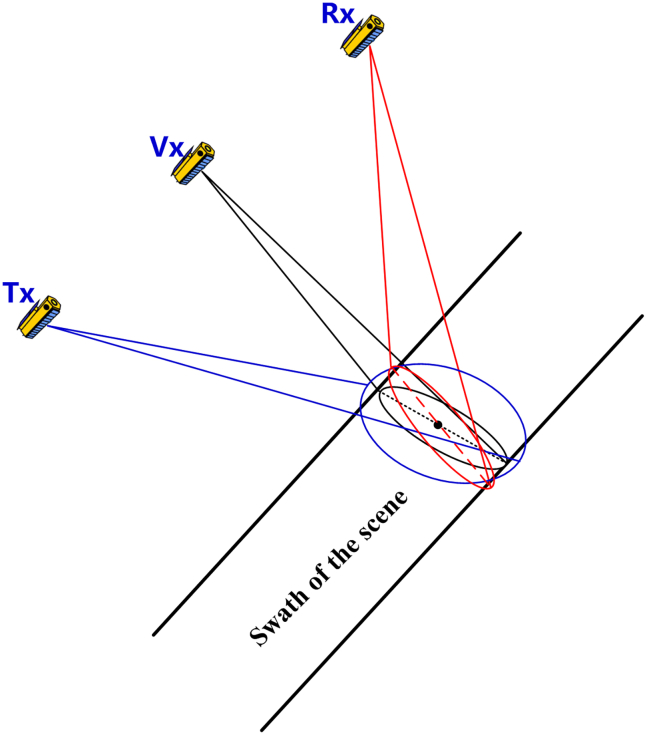
The illustration of the implementation of the 2^nd^ attitude steering strategy

The implementation of this strategy is illustrated by Figure 8, the bistatic Doppler frequency along the range line of the receiving antenna beam footprint, denoted by the dashed red line, will be kept as close to zero as possible.

### Attitude steering based on the transmitting satellite and footprint overlapping

In the 3^rd^ attitude steering strategy for distributed spaceborne SAR, its basic rationale and procedures are identical to those of the 1^st^ strategy. The differences between them are that the virtual satellite is not necessary anymore in this strategy, since the transmitting satellite is employed as the reference satellite and the beam footprint of the receiving satellite should be adjusted to overlap with it as much as possible.

**Figure 9:**
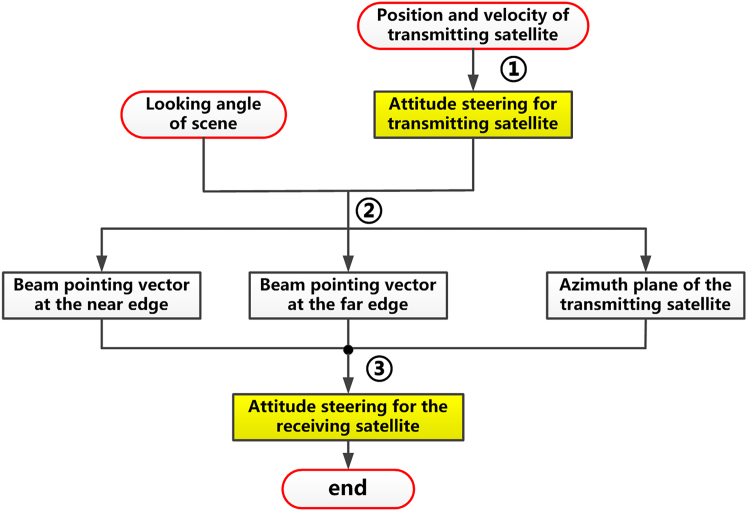
The flowchart of the 3^rd^ attitude steering strategy for distributed spaceborne SAR

Figure 9 presents the workflow of this attitude steering strategy. It can be seen that the attitude steering for the transmitting satellite is the same as that used for the conventional spaceborne SAR. Then, the attitude of receiving the satellite is steered to achieve the goal of overlapping beam footprints.

### Attitude steering based on transmitting satellite and bistatic Doppler centroid

This attitude steering strategy is similar to that of the 2^nd^ strategy, and their difference is that the transmitting satellite, rather than the virtual satellite, is used as the reference satellite, and the attitude of the receiving satellite is designed in such a way that the bistatic Doppler frequency will not vary along the range line of the receiving antenna beam footprint.

**Figure 10:**
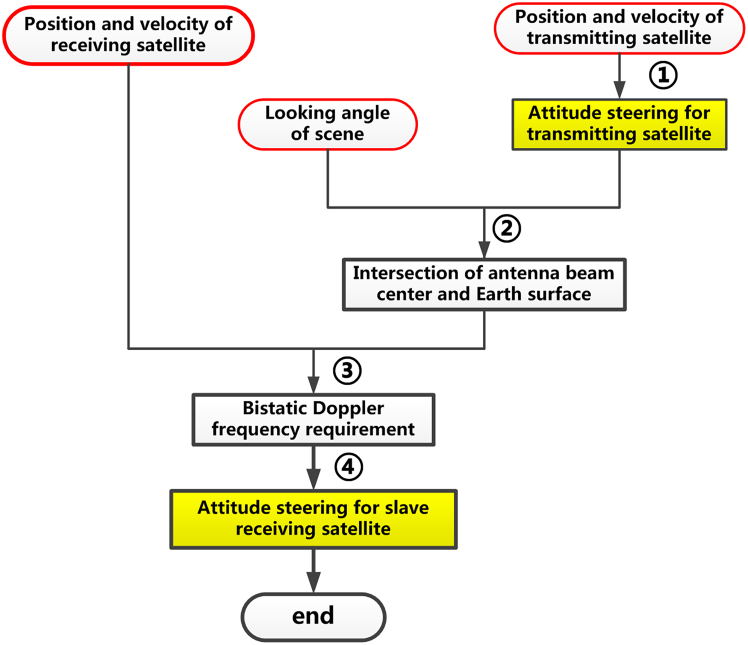
The flowchart of the 4^th^ attitude steering strategy for distributed spaceborne SAR

In this strategy, the attitude steering can be implemented in four steps, as shown in Figure 10.

Step 1: Determine the attitude steering strategy of the transmitting satellite

The attitude of the transmitting satellite is designed in the same way as the conventional spaceborne SAR.

Step 2: Determine the intersection position of the antenna beam center of the transmitting satellite and the Earth’s surface

Based on the attitude of the transmitting satellite, its unit vectors of the three axes ${\hat{x}}_{\text{Tx}}$, ${\hat{y}}_{\text{Tx}}$, and ${\hat{z}}_{\text{Tx}}$ can then be determined. Then, the intersection of vector ${\hat{z}}_{\text{Tx}}$ and the Earth’s surface can be obtained using the following three equations

(Equation 57) $$\left({\vec{p}}_{c}-{\vec{s}}_{\text{Tx}}\right)\cdot {\hat{x}}_{\text{Tx}}=0$$

(Equation 58) $$\left({\vec{p}}_{c}-{\vec{s}}_{\text{Tx}}\right)\cdot {\hat{y}}_{\text{Tx}}=0$$

(Equation 60) $${\left[{\vec{p}}_{c}\left(x\right)\right]}^{2}/R_{a}^{2}+{\left[{\vec{p}}_{c}\left(y\right)\right]}^{2}/R_{a}^{2}+{\left[{\vec{p}}_{c}\left(z\right)\right]}^{2}/R_{b}^{2}=1$$

where ${\vec{p}}_{c}$ represents this intersection, and ${\vec{p}}_{c}\left(x\right)$, ${\vec{p}}_{c}\left(y\right)$, and ${\vec{p}}_{c}\left(z\right)$ are its three components projected to the X-, Y-, and z axis, respectively.

Step 3: Determine the bistatic Doppler frequency requirement

With the obtained position of the intersection ${\vec{p}}_{c}$, along with the position and velocity knowledge of transmitting and receiving satellites, its bistatic Doppler frequency *f*_bi_ can be determined as

(Equation 61) $$f_{\text{bi}}=-\frac{\left({\vec{p}}_{c}-{\vec{s}}_{\text{Tx}}\right)}{\lambda \left\|{\vec{p}}_{c}-{\vec{s}}_{\text{Tx}}\right\|}\cdot {\vec{v}}_{\text{Tx}}-\frac{\left({\vec{p}}_{c}-{\vec{s}}_{\text{Rx}}\right)}{\lambda \left\|{\vec{p}}_{c}-{\vec{s}}_{\text{Rx}}\right\|}\cdot {\vec{v}}_{\text{Rx}}$$

Step 4: Determine the attitude steering strategy of receiving satellite

In this step, it follows the same derivation procedures as given in Step 5 of the 2^nd^ attitude steering strategy. And the only difference between them is that the bistatic Doppler frequency should be equal to *f*_bi_ given in Equation 61 rather than zero in this strategy.

## Results

In this section, we will employ a general distributed spaceborne SAR system comprised of a master transmitting satellite and a slave receiving satellite. They are on the same orbital plane and have a time interval between them. Here, we assume that the transmitting satellite is Δ*T* = 0.7*s* after the receiving satellite, as shown in Figure 5. The orbit and system simulation parameters are listed in Table 1.

**Table 1:** Orbit and system parameters for the attitude steering of distributed spaceborne SAR

Index	Parameter	Value
1	mean semi-axis of the orbit	6871 km
2	mean eccentricity of the orbit	1.5 × 10^−3^
3	mena inclination of the orbit	97.39°
4	right ascension of ascending node (RAAN)	179.52°
5	Azimuth beamwidth of the transmitting antenna	1°
6	Azimuth beamwidth of the receiving antenna	1°
7	elevation beamwidth of the transmitting antenna	2°
8	elevation beamwidth of the receiving antenna	2°
9	SAR carrier frequency	9.8 GHz
10	time interval between transmitting and receiving the satellite	0.7 s
11	looking angle of the scene center	30°

**Figure 11:**
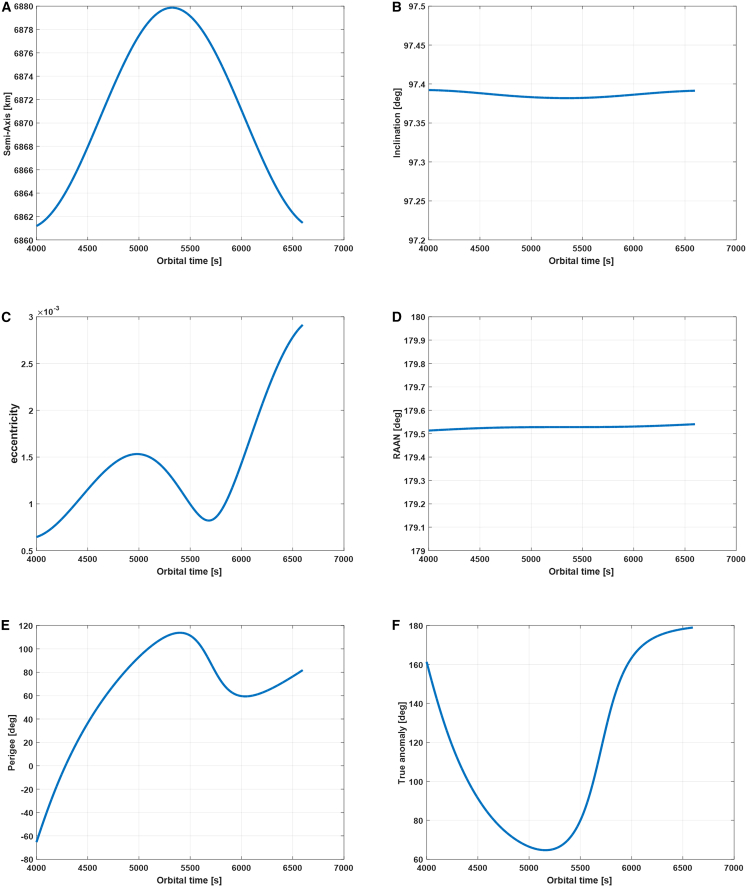
The corresponding parameters of the orbit data that are to be used for the derivation of attitude steering of the distributed spaceborne SAR system (A) semi-axis, (B) inclination, (C) eccentricity, (D) RAAN, (E) perigee, and (F) true anomaly.

Here, we slice a section of orbit data of a spaceborne SAR satellite, and its time length is 2600s. The corresponding orbital parameters within this period, including the semi-axis, inclination, eccentricity, right ascension of ascending node, perigee, and true anomaly, are presented in Figure 11. This satellite operates on a low earth orbiting (LEO) orbit with a fixed right ascension of ascending node (RAAN) value. Its orbital model can be considered as a two-body model with J2-perturbation, which is compensated by orbit maneuvering. From subfigure (d) of Figure 11, it can be seen that RAAN has always remained constant to avoid the drifting of the orbital plane.

**Figure 12:**
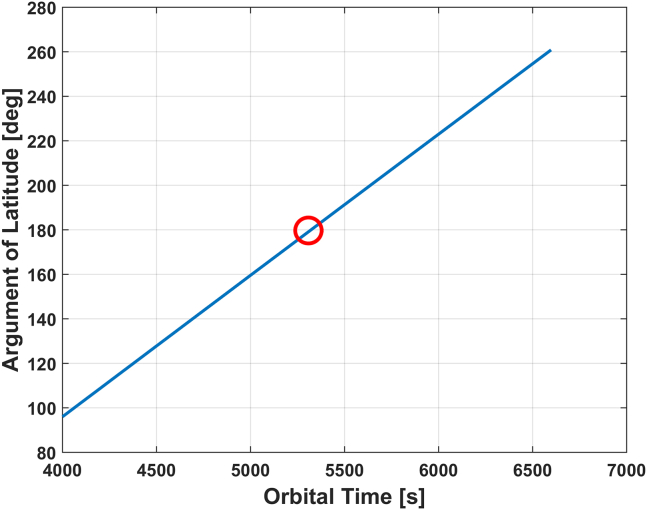
Argument of latitude of the satellite during the sliced orbital period

With the above orbital elements, we can obtain the corresponding argument of latitude of the transmitting satellite during this period, as shown in Figure 12. It can be seen that the satellite will be crossing the equatorial plane at T ≈ 5325 s (denoted by the red circle).

### Attitude steering simulation results

**Figure 13:**
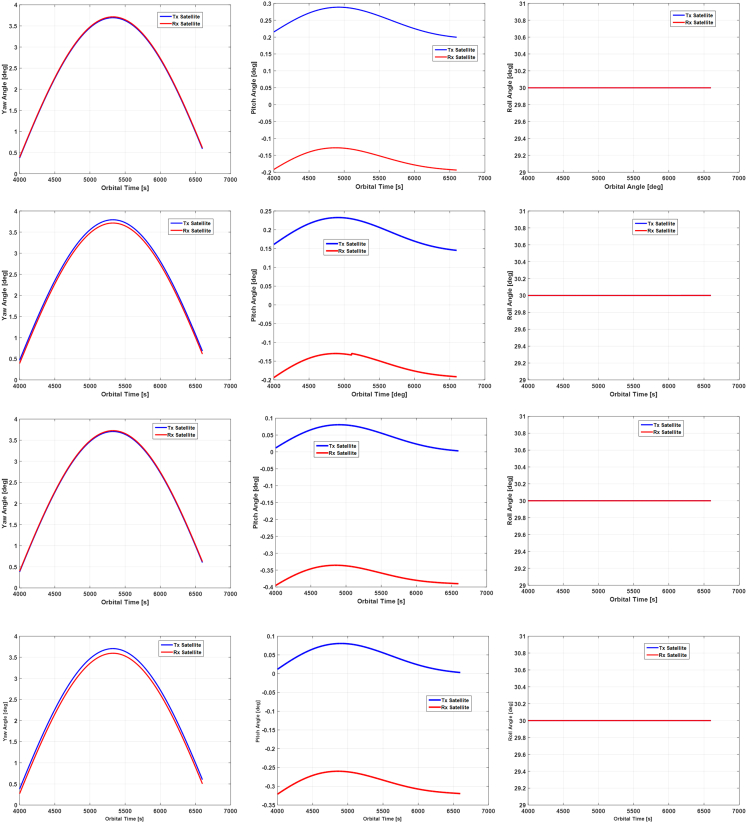
The required Euler angles of transmitting and receiving satellites during the 2600s orbital period The topmost to the bottom rows correspond to the 1^st^ ∼ 4^th^ attitude steering strategy, respectively.

In this section, we will present the simulation results of Euler angles corresponding to four different attitude steering strategies, as shown in Figure 13.

From the above shown results, it can be seen that the closer to the equatorial plane the satellite moves, the higher its yaw angle will become. Compared with pitch and roll, the yaw angle varies more significantly with the argument of latitude. Its highest value has achieved almost 4° while its minimum value is about 0.5°. Since both the first and the second strategies introduce the virtual satellite when deriving the Euler angles, their corresponding results will be compared first. From the 1^st^ and 2^nd^ rows of Figure 13, we can see that the yaw angles of the transmitting satellite for these two cases are almost identical, while the pitch angle decreases a little bit from the 1^st^ to the 2^nd^ strategy. Similarly, by comparing results in the 3^rd^ and the 4^th^ row of Figure 13, it can be observed that attitudes of the transmitting satellite in these two strategies are identical since it is used as the reference satellite, while the yaw and the pitch angle of the receiving satellite are reduced when the strategy changes from the 3^rd^ to the 4^th^ one.

In the next subsection, we will analyze the Doppler frequency results and their variation along the range line of the reference satellite.

### Doppler frequency simulation results

**Figure 14:**
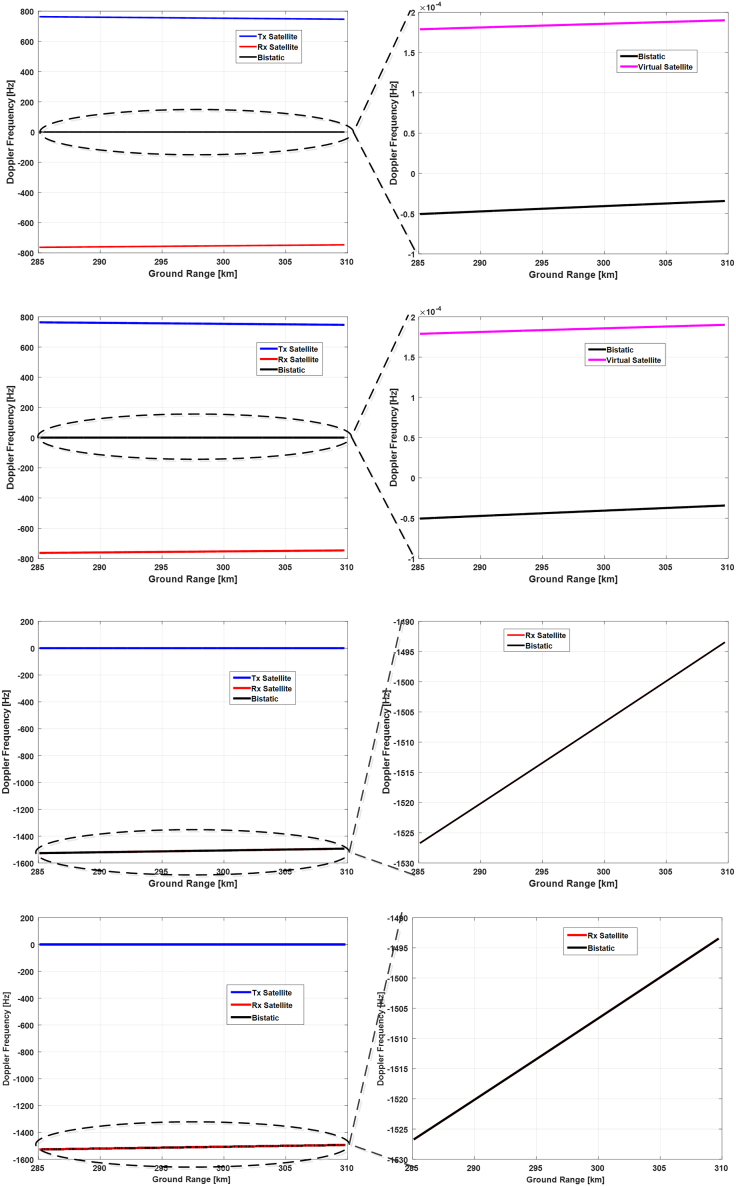
Doppler frequencies along the range lines of reference antenna beam at the starting time of the selected orbital period The results from the topmost to the bottom row correspond to the 1^st^ to the 4^th^ attitude steering strategy. The left column shows the Doppler frequencies of transmitting (blue) and receiving satellite (red), along with the bistatic Doppler frequency (black), and the right column gives the zoom-in of the bistatic Doppler frequency and compares it with the virtual (pink) or receiving Doppler frequency (red).

**Figure 15:**
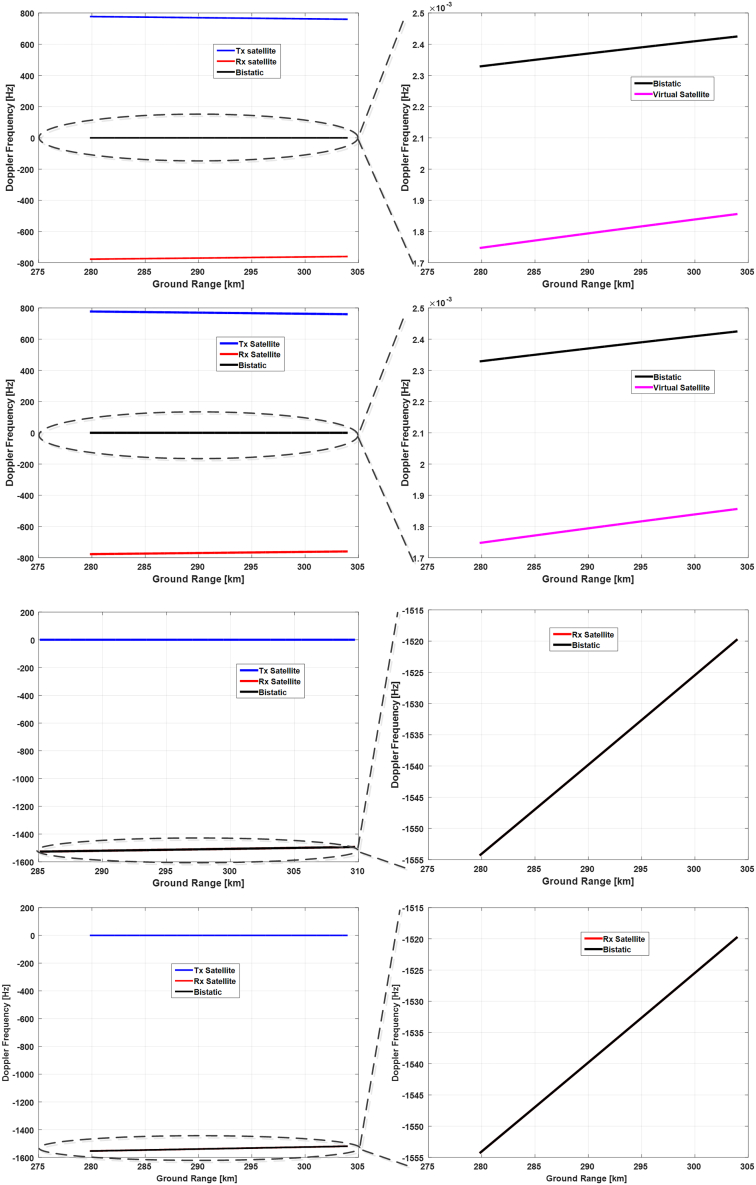
Doppler frequencies along the range lines of reference antenna beam at the time of the yaw angle peak within the selected orbital period

**Figure 16:**
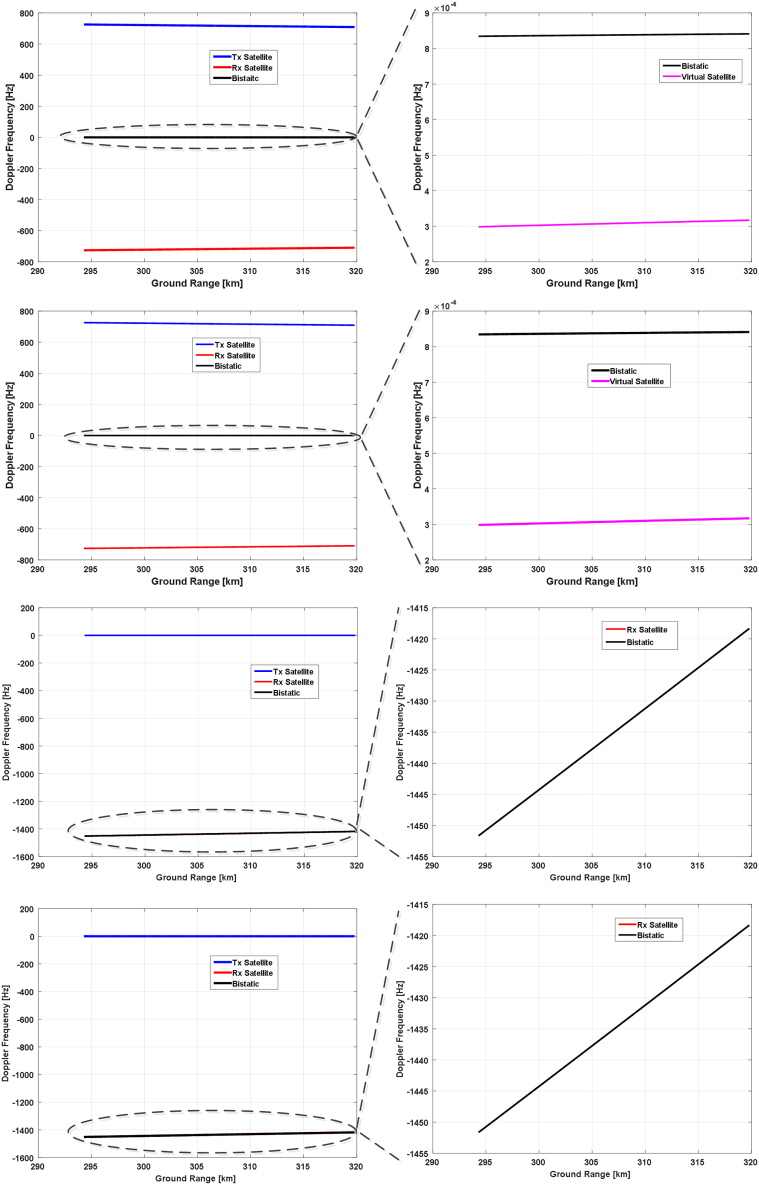
Doppler frequencies along the range lines of reference antenna beam at the end of the selected orbital period

Utilizing the Euler angle results obtained in the last subsection, we will analyze the Doppler frequency characteristics realized by these attitude steering strategies. Three representative time instants within the selected orbital period, namely the starting time, the ending time, and the time that the yaw angle achieves its highest value, will be chosen to simulate the Doppler frequency and analyze their properties. Corresponding results are presented in Figures 14, 15, and 16, respectively.

At first, we compare the results in the 1^st^ and the 2^nd^ row of Figure 14, and we can observe that they have obtained almost the same bistatic Doppler frequency, although with different Euler angles. And the bistatic Doppler frequency is very close to zero and varies barely in the range direction, and can thus be neglected safely.

Next, the results in the 3^rd^ and the 4^th^ row are compared since both of them have adopted the transmitting satellite as a reference. It can be observed that these two strategies have also realized almost identical results, thereby validating the effectiveness of the attitude steering strategy employing either the virtual satellite or the transmitting satellite as a reference in terms of bistatic Doppler frequency. Concerning the last two strategies, the attitude of the transmitting satellite is steered with the conventional yaw-pitch steering strategy, and thus its Doppler frequency will be close to zero and vary slightly along the range line. Consequently, the property of bistatic Doppler frequency is mainly determined by the receiving Doppler frequency. Since the pitch angle of the receiving satellite in these two strategies is almost twice that of the first two ones, the Doppler frequency variation has become larger and achieved almost 35 Hz.

Then, Doppler frequency simulation results at the time of the highest yaw angle and at the ending time are presented in Figures 15 and 16, respectively. It can be seen that the bistatic Doppler frequency, as well as its variation along the range line, becomes almost closer to zero for the 1^st^ and the 2^nd^ strategy. Besides, the bistatic Doppler frequency obtained by the strategies employing the virtual satellite as reference has much smaller values, and its variation along the range line can be neglected as compared to that utilizing the transmitting satellite as reference.

### Antenna beam footprints simulation results

**Figure 17:**
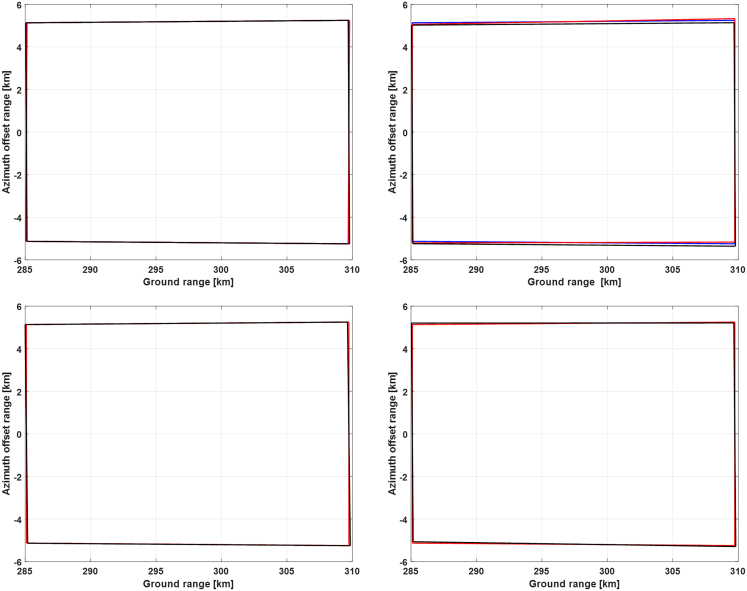
Antenna beam footprints of different attitude steering strategies at the starting time of the selected orbital period (Upper left) results of the 1^st^ strategy, the area defined by the blue, red and black lines denote the beam footprints of the virtual, the transmitting and the receiving satellite respectively; (upper right) results of the 2^nd^ strategy; (lower left) results of the 3^rd^ strategy, where the virtual satellite is not needed anymore; (lower right) results of the 4^th^ strategy.

**Figure 18:**
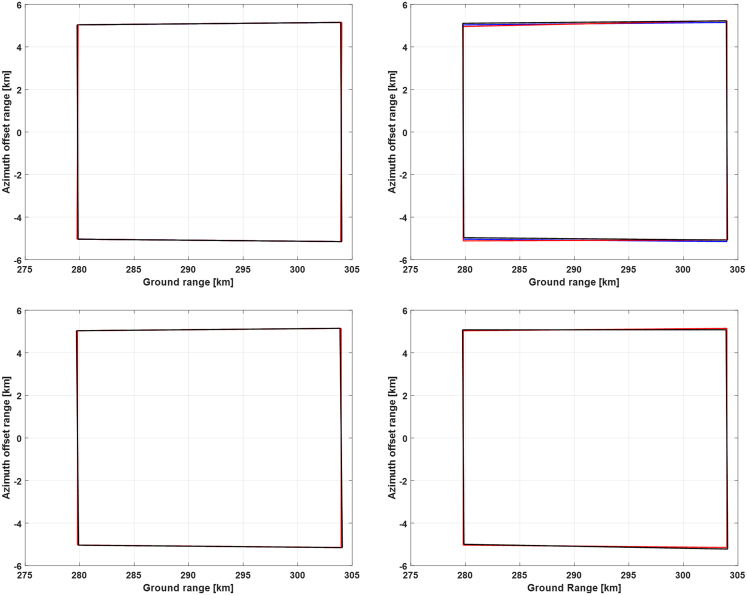
Antenna beam footprints of different attitude steering strategies at the time of the yaw angle peak within the selected orbital period The same sequence and the same colored lines as above are used to show results corresponding to these four different attitude steering strategies.

**Figure 19:**
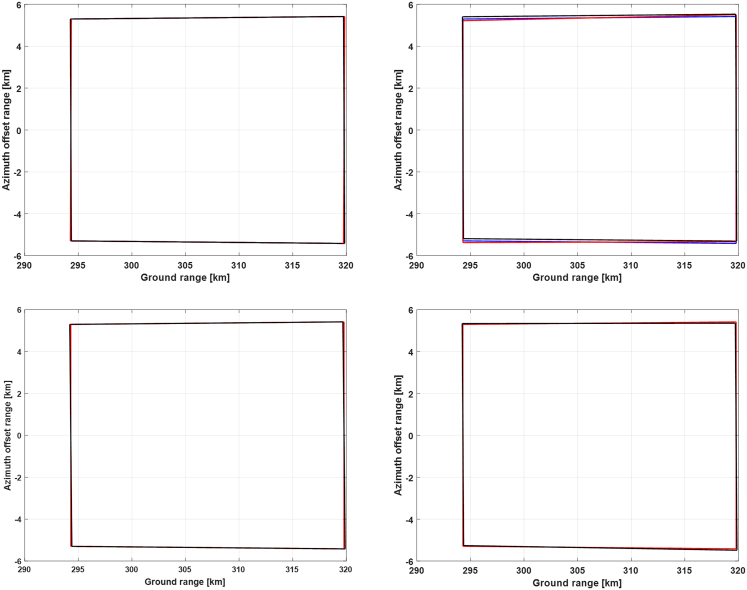
Antenna beam footprints of different attitude steering strategies at the end of the selected orbital period The same sequence and the same colored lines as above are used to show results corresponding to these four different attitude steering strategies.

At last, we will analyze the coverage of antenna beam footprints of different attitude steering strategies. Euler angles at the above three representative time instants are still selected to simulate the transmitting and receiving antenna beam footprints. Figures 17, 18, and 19 show the corresponding coverage of antenna beams at these three-time instants. In order to quantify and compare the coverage of each strategy, we employ the metrics intersection over union (IoU) can to calculate coverage precisely, enabling an objective and numerical comparison of differences between strategies. Tables 2, 3, and 4 present the beam footprint coverage area of both transmitting and receiving satellites, as well as their area of intersection and area of union. In this way, the IoU can be derived to show and compare their performances clearly.

**Table 2:** Footprint coverage analysis and comparison at the starting time of the selected orbital period

Strategy	Area of Tx beam footprint	Area of Rx beam footprint	Area of Intersection	Area of Union	Intersection of Union (IoU)
1	255.6119 km^2^	255.6128 km^2^	255.4739 km^2^	255.7508 km^2^	99.8917%
2	255.6114 km^2^	255.6129 km^2^	255.0843 km^2^	256.1400 km^2^	99.5878%
3	255.6102 km^2^	255.6236 km^2^	255.4778 km^2^	255.7560 km^2^	99.8912%
2	255.6102 km^2^	255.6217 km^2^	255.2353 km^2^	255.9967 km^2^	99.7026%

**Table 3:** Footprint coverage analysis and comparison at the time of the yaw angle peak within the selected orbital period

Strategy	Area of Tx beam footprint	Area of Rx beam footprint	Area of Intersection	Area of Union	Intersection of Union (IoU)
1	246.2493 km^2^	246.2466 km^2^	246.1117 km^2^	246.3842 km^2^	99.8894%
2	246.2488 km^2^	246.2466 km^2^	246.7551 km^2^	246.7403 km^2^	99.6007%
3	246.2424 km^2^	246.2522 km^2^	246.1111 km^2^	246.3835 km^2^	99.8894%
2	246.2424 km^2^	246.2503 km^2^	245.8731 km^2^	246.6196 km^2^	99.6973%

**Table 4:** Footprint coverage analysis and comparison at the end of the selected orbital period

Strategy	Area of Tx beam footprint	Area of Rx beam footprint	Area of Intersection	Area of Union	Intersection of Union (IoU)
1	272.9676 km^2^	272.9653 km^2^	272.8226 km^2^	273.1103 km^2^	99.8947%
2	272.9671 km^2^	272.9652 km^2^	272.4524 km^2^	273.4800 km^2^	99.6243%
3	272.9613 km^2^	272.9714 km^2^	272.8255 km^2^	273.1102 km^2^	99.8947%
2	272.9613 km^2^	272.9695 km^2^	272.5753 km^2^	273.3555 km^2^	99.7146%

By comparing the beam footprints coverage of these four strategies at three representative time instants, one can observe that the strategy that focuses on the beam footprints overlapping (strategies 1 and 3) has obtained slightly better performance than that defined by the bistatic Doppler frequency along the range line (strategies 2 and 4), as demonstrated by the last column of Tables 2, 3, and 4. The footprints' offsets will cause certain SNR degradation at the edge of the observed scene, since the optimal two-way antenna gain cannot be obtained. Nevertheless, since the values of IoU corresponding to the four strategies have all been above 99.5%, the gain or loss due to the non-overlapping of beam footprints can be neglected safely.

## Discussion

Based on the simulation results acquired in the last subsection, we can tentatively get the following conclusions.

1. The attitude steering strategies that introduce the virtual satellite as the reference will make sure that the bistatic Doppler frequency is identical to that of the virtual satellite using conventional yaw-pitch steering rules, and thus it will facilitate the imaging processing.
2. The variation of bistatic Doppler frequencies associated with the 1^st^ and the 2^nd^ strategies is quite small and can be safely neglected.
3. The 1^st^ strategy can achieve slightly better performance in terms of beam footprints overlapping than that of the 2^nd^ strategy.
4. The bistatic Doppler frequency of the strategies that are based on the Tx satellite has much larger values, and its variation along the range line can achieve as high as 35 Hz.
5. For the last two strategies, their performances are almost identical to each other in terms of either bistatic Doppler frequency or beam footprints overlapping.

On the basis of attitude steering for the conventional spaceborne SAR on a single platform, our work focused on the attitude steering scheme for the future distributed spaceborne SAR, and presented a total of four different strategies as well as the detailed procedures for each of them. Compared to the attitude steering of a single platform, our strategies have taken into account both the transmitting and receiving satellites, including their position and velocity, to make sure that their beam footprints overlap as much as possible and that the Doppler frequency of received bistatic echoes be kept constant along the range line to the greatest extent at the same time.

Through simulation results, it can be seen that all of these four strategies have achieved satisfactory overlapping of beam footprints. Moreover, by introducing the concept of a virtual satellite, the 1^st^ and the 2^nd^ strategies can realize the goal that the bistatic Doppler frequency along the range line could be very close to zero. As for the 3^rd^ and the 4^th^ strategies, the bistatic Doppler frequency is determined dominantly by the receiving satellite, and its variation along the range line has achieved 35 Hz, which can also be neglected safely for the vast majority of spaceborne SAR processing and applications.

From the perspective of efficiency and precision, the 1^st^ and the 3^rd^ strategies will be much better, as all the required positions in the whole process of attitude derivation are determined decisively via the equations and can be expressed explicitly. Nevertheless, as for the 2^nd^ and the 4^th^ strategies, their procedures will involve the process of position searching within certain azimuthal planes, as their equations cannot be solved directly (variables exist in both of the nominator and denominator of equations, as shown in (Equation 47), (Equation 61)), it will be more time-consuming to determine the position values and there will also be certain deviations of these values from the real positions, and these deviation values are determined by the gaps of search grid within the planes.

In the end, we summarize the optimal application scenarios for these attitude steering strategies.

1. If the whole distributed spaceborne SAR system consists of one transmitting and one receiving satellite, and both of their platform attitudes can be maneuvered agilely, the 1^st^ strategy is the most appropriate solution.
2. If there are still two satellites in the whole system, but the attitude steering capability and of transmitting satellite, as the master satellite, is limited and cannot be maneuvered swiftly and precisely like the receiving satellite (slave satellite), the 3^rd^ strategy should be adopted.
3. For the future distributed system involving one transmitting but multiple receiving satellites, the virtual satellite cannot be determined. In this case, the 3^rd^ strategy is the only optimal solution.

### Limitations of the study

In this work, the distributed spaceborne SAR system is assumed to be in a bistatic mode, which consists of one transmitting and one receiving satellite. Thus, it takes only these two satellites into consideration when designing the attitude steering strategy and the system model can be simplified to a certain extent. Nevertheless, the future distributed SAR system might be comprised of several transmitting as well as receiving satellites. In this scenario, the positions and velocities of all these satellites should be taken into account. And the attitude derivation procedure for all these satellites might be more complex, and several trade-offs should be made to achieve an optimal balance among different factors. Therefore, our research work will then be focused on multiple input multiple output (MIMO) distributed spaceborne SAR system to make a deeper insight into this issue.

## Resource availability

### Lead contact

Requests for further information and resources should be directed to and will be fulfilled by the lead contact, Haifeng Yu (sailingvon2023@163.com).

### Materials availability

This study did not generate new unique reagents.

### Data and code availability

• Data reported in this paper will be shared by the lead contact upon request.
• This paper does not report original code.
• Any additional information required to reanalyze the data reported in this paper is available from the lead contact upon request.

## Acknowledgments

This work is supported by the Foundation of 10.13039/501100008114Nanjing Institute of Technology under the grant no. YKJ202362.

## Author contributions

Conceptualization, H. Y.; methodology, H. Y.; investigation, H.Y., writing – original draft, H. Y.; writing – review and editing, H. Y.; funding acquisition, H. Y.; resources, H. Y.; supervision, H. Y.

## Declaration of interests

The author declares no competing interests.

## STAR★Methods

### Key resources table

**Table:** 

REAGENT or RESOURCE	SOURCE	IDENTIFIER
**Software and algorithms**		

MATLAB 2015a	MathWorks	https://www.mathworks.com/matlabcentral/
Systems Tool Kit (STK)	Analytical Graphics INC.(AGI)	http://www.agi.com/support.

### Experimental model and study participant details

Our study does not use experimental models typical in life science.

### Method details

As discussed above, the 1^st^ attitude steering strategy is the most appropriate option for the distributed spaceborne SAR. Then, we will make a detailed description of its key derivation steps.

#### Derivation of three unit vectors of satellite body coordinates from Euler angles

From Euler angles and the associated rotation sequence (Yaw-Pitch-Roll in our study), three unit vectors corresponding to three axes of the satellite body coordinates can be derived from Equation 62.

(Equation 62) $$\left[X_{1};Y_{1};Z_{1}\right]=M_{\text{roll}}\cdot M_{\text{pitch}}\cdot M_{\text{yaw}}$$

where **M**_yaw_, **M**_pitch_ and **M**_roll_ are rotation matrixes corresponding to yaw, pitch, and roll maneuvers. And their explicit expressions are

(Equation 63) $$M_{\text{yaw}}=\left[\begin{array}{ccc} \cos\;\theta_{\text{yaw}} & \sin\;\theta_{\text{yaw}} & 0\\ -\sin\;\theta_{\text{yaw}} & \cos\;\theta_{\text{yaw}} & 0\\ 0 & 0 & 1 \end{array}\right]$$

(Equation 64) $$M_{\text{pitch}}=\left[\begin{array}{ccc} \cos\;\theta_{\text{pitch}} & 0 & -\sin\;\theta_{\text{pitch}}\\ 0 & 1 & 0\\ \sin\;\theta_{\text{pitch}} & 0 & \cos\;\theta_{\text{pitch}} \end{array}\right]$$

(Equation 65) $$M_{\text{roll}}=\left[\begin{array}{ccc} 1 & 0 & 0\\ 0 & \cos\;\theta_{\text{roll}} & \sin\;\theta_{\text{roll}}\\ 0 & -\sin\;\theta_{\text{roll}} & \cos\;\theta_{\text{roll}} \end{array}\right]$$

respectively. Then, with the satellite position $P_{\text{SAT}\_\text{ECEF}}$ and velocity $V_{\text{SAT}\_\text{ECEF}}$ of the virtual satellite defined in the Earth-Centered Earth Fixed (ECEF) coordinate frame, we can directly obtain three unit vectors of orbit coordinate **U**_Z_, **U**_Y_ and **U**_X_ as

(Equation 66) $$U_{Z}=-P_{\text{SAT}\_\text{ECEF}}/\left\|P_{\text{SAT}\_\text{ECEF}}\right\|$$

(Equation 67) $$V_{U}=V_{\text{SAT}\_\text{ECEF}}+\omega_{e}\times P_{\text{SAT}\_\text{ECEF}}$$

(Equation 68) $$U_{Y}=\left(U_{Z}\times V_{U}\right)/\left\|U_{Z}\times V_{U}\right\|$$

(Equation 69) $$U_{X}=U_{Z}\times U_{Y}$$

where ω_*e*_ is the self-rotation angular velocity of Earth. The intermediate variable **V**_U_ is the inertial satellite velocity. Then, the transformation matrix from the orbit coordinates to the ECEF coordinates is given by

(Equation 70) $$M_{\text{orb}\to \text{ECEF}}={\left[U_{X};U_{Y};U_{Z}\right]}^{T}$$

With results given by (Equation 62), (Equation 70), we can finally obtain three unit vectors of satellite body coordinates defined in the Earth-Centered Earth-Fixed (ECEF) coordinate frame as

(Equation 71) $$\left[{\hat{X}}_{v};{\hat{Y}}_{v};{\hat{Z}}_{v}\right]=M_{\text{orb}\to \text{ECEF}}\left[X_{1};Y_{1};Z_{1}\right]$$

#### Determination of the intersection point between the z axis of satellite and the Earth’s surface

In Step 4.3 of the 1^st^ attitude steering strategy, the precondition for the determination of z axis of the transmitting satellite is to obtain its intersection with the Earth’s surface. The detailed derivation process from (Equation 24), (Equation 25), (Equation 26) is given below.

To determine this intersection point, a series of intermediate variables should be introduced to decrease the computational complexity.

#### Define

(Equation 72) $$k_{1}=\left[{\vec{y}}_{v}\left(1\right)\cdot {\vec{x}}_{\text{Tx}}\left(3\right)-{\vec{y}}_{v}\left(3\right)\cdot {\vec{x}}_{\text{Tx}}\left(1\right)\right]/\left[{\vec{x}}_{\text{Tx}}\left(2\right)\cdot {\vec{y}}_{v}\left(3\right)-{\vec{y}}_{v}\left(2\right)\cdot {\vec{x}}_{\text{Tx}}\left(3\right)\right]$$

(Equation 73) $$d_{1}=\left[{\vec{x}}_{\text{Tx}}\left(1\right){\vec{y}}_{v}\left(3\right){\vec{s}}_{\text{Tx}}\left(1\right)+{\vec{x}}_{\text{Tx}}\left(2\right){\vec{y}}_{v}\left(3\right){\vec{s}}_{\text{Tx}}\left(2\right)+{\vec{y}}_{v}\left(2\right){\vec{x}}_{\text{Tx}}\left(3\right){\vec{s}}_{\text{Tx}}\left(3\right)\right]/\left[{\vec{x}}_{\text{Tx}}\left(2\right){\vec{y}}_{v}\left(3\right)-{\vec{x}}_{\text{Tx}}\left(3\right){\vec{y}}_{v}\left(2\right)\right]-\left[{\vec{x}}_{\text{Tx}}\left(3\right){\vec{y}}_{v}\left(1\right){\vec{s}}_{\text{vx}}\left(1\right)+{\vec{x}}_{\text{Tx}}\left(3\right){\vec{y}}_{v}\left(2\right){\vec{s}}_{\text{vx}}\left(2\right)+{\vec{y}}_{v}\left(3\right){\vec{x}}_{\text{Tx}}\left(3\right){\vec{s}}_{\text{vx}}\left(3\right)\right]/\left[{\vec{x}}_{\text{Tx}}\left(2\right){\vec{y}}_{v}\left(3\right)-{\vec{x}}_{\text{Tx}}\left(3\right){\vec{y}}_{v}\left(2\right)\right]$$

(Equation 74) $$k_{2}=\left[{\vec{y}}_{v}\left(1\right)\cdot {\vec{x}}_{\text{Tx}}\left(2\right)-{\vec{y}}_{v}\left(2\right)\cdot {\vec{x}}_{\text{Tx}}\left(1\right)\right]/\left[{\vec{x}}_{\text{Tx}}\left(3\right)\cdot {\vec{y}}_{v}\left(2\right)-{\vec{y}}_{v}\left(3\right)\cdot {\vec{x}}_{\text{Tx}}\left(2\right)\right]$$

(Equation 75) $$d2=\left[{\vec{x}}_{\text{Tx}}\left(1\right){\vec{y}}_{v}\left(2\right){\vec{s}}_{\text{Tx}}\left(1\right)+{\vec{x}}_{\text{Tx}}\left(2\right){\vec{y}}_{v}\left(2\right){\vec{s}}_{\text{Tx}}\left(2\right)+{\vec{y}}_{v}\left(2\right){\vec{x}}_{\text{Tx}}\left(3\right){\vec{s}}_{\text{Tx}}\left(3\right)\right]/\left[{\vec{x}}_{\text{Tx}}\left(3\right){\vec{y}}_{v}\left(2\right)-{\vec{x}}_{\text{Tx}}\left(2\right){\vec{y}}_{v}\left(3\right)\right]-\left[{\vec{x}}_{\text{Tx}}\left(2\right){\vec{y}}_{v}\left(1\right){\vec{s}}_{\text{vx}}\left(1\right)+{\vec{x}}_{\text{Tx}}\left(2\right){\vec{y}}_{v}\left(2\right){\vec{s}}_{\text{vx}}\left(2\right)+{\vec{y}}_{v}\left(3\right){\vec{x}}_{\text{Tx}}\left(2\right){\vec{s}}_{\text{vx}}\left(3\right)\right]/\left[{\vec{x}}_{\text{Tx}}\left(3\right){\vec{y}}_{v}\left(2\right)-{\vec{x}}_{\text{Tx}}\left(2\right){\vec{y}}_{v}\left(3\right)\right]$$

Then, we can obtain three intermediate variables from the above quantity as

(Equation 76) $$a_{0}=1/R_{a}^{2}+k_{1}^{2}/R_{a}^{2}+k_{2}^{2}/R_{b}^{2}$$

(Equation 77) $$b_{0}=2k_{1}d_{1}/R_{a}^{2}+2k_{2}d_{2}/R_{b}^{2}$$

(Equation 78) $$c_{0}=d_{1}^{2}/R_{a}^{2}+d_{2}^{2}/R_{b}^{2}-1$$

Finally, three components of this intersection point on Earth’s surface can be obtained with (Equation 72), (Equation 73), (Equation 74), (Equation 75), (Equation 76), (Equation 77), (Equation 78) as

(Equation 79) $$X_{\text{point}}=\left(-b+\sqrt{b^{2}-4ac}\right)/2a$$

(Equation 80) $$Y_{\text{point}}=k_{1}X+d_{1}$$

(Equation 81) $$Z_{\text{point}}=k_{2}X+d_{2}$$

#### Derivation of Euler angles from three unit vectors of satellite body in the ECEF frame

From Equation 70, we can obtain the transformation matrix from the ECEF frame to the orbit frame as the transposition of matrix $M_{\text{orb}\to \text{ECEF}}$ and it can be expressed as

(Equation 82) $$M_{\text{ECEF}\to \text{orb}}=\left[U_{X};U_{Y};U_{Z}\right]$$

Then, three unit vectors of the satellite body can be defined in the orbit coordinates from the original ECEF coordinates as

(Equation 83) $$\left[X_{1};Y_{1};Z_{1}\right]=M_{\text{ECEF}\to \text{orb}}\left[{\hat{X}}_{v};{\hat{Y}}_{v};{\hat{Z}}_{v}\right]$$

With the matrix given by [**X**_1_;**Y**_1_;**Z**_1_], three Euler angles defined in the Yaw-Pitch-Rotation rotation sequence can be obtained as

(Equation 84) $$\theta_{\text{pitch}}=-\arcsin\left[X_{1}\left(3\right)\right]$$

(Equation 85) $$\theta_{\text{roll}}=\arcsin\left[Y_{1}\left(3\right)/\cos\left(\theta_{\text{pitch}}\right)\right]$$

(Equation 86) $$\theta_{\text{roll}}=\arcsin\left[Y_{1}\left(3\right)/\cos\left(\theta_{\text{pitch}}\right)\right]$$

### Quantification and statistical analysis

In our study, orbital elements of the transmitting and receiving satellite are obtained from an in-orbit SAR satellite. And velocities and positions of these two satellites, which are defined within the Earth-Centered Earth-Fixed (ECEF) coordinate frame, are derived from these orbital elements by STK software. Then, the bistatic Doppler frequencies as well as the beam footprints overlapping are simulated and analyzed by MATLAB software.

## References

[1] Massonnet D. (2001). Capabilities and limitations of the interferometric cartwheel. IEEE Trans. Geosci. Remote Sens..

[2] Krieger G., Hajnsek I., Papathanassiou K.P., Younis M., Moreira A. (2010). Interferometric synthetic aperture radar (SAR) missions employing formation flying. Proc. IEEE.

[3] Zink M., Moreira A., Hajnsek I., Rizzoli P., Bachmann M., Kahle R., Fritz T., Huber M., Krieger G., Lachaise M. (2021). TanDEM-X: 10 years of formation flying bistatic SAR interferometry. IEEE J. Sel. Top. Appl. Earth Obs. Remote Sens..

[4] Hajnsek I., Busche T., Abdullahi S., Bachmann M., Baumgartner S.V., Bojarski A., Bueso-Bello J.L., Esch T., Fritz T., Alonso-Gonzalez A. (2025). TanDEM-X: The 4D Mission Phase for Earth Surface Dynamics: Science activities highlights and new data products after 15 years of bistatic operations. IEEE Geosci. Remote Sens. Mag..

[5] Esch T., Brzoska E., Dech S., Leutner B., Palacios-Lopez D., Metz-Marconcini A., Marconcini M., Roth A., Zeidler J. (2022). World settlement footprint 3D-A first three-dimensional survey of the global building stock. Remote Sens. Environ..

[6] Martone M., Rizzoli P., Wecklich C., González C., Bueso-Bello J.L., Valdo P., Schulze D., Zink M., Krieger G., Moreira A. (2018). The global forest/non-forest map from TanDEM-X interferometric SAR data. Remote Sens. Environ..

[7] Bueso-Bello J.-L., Carcereri D., Martone M., González C., Posovszky P., Rizzoli P. (2022). Deep Learning for Mapping Tropical Forests with TanDEM-X Bistatic InSAR Data. Remote Sens..

[8] Soja M.J., Persson H., Ulander L.M.H. (2015). Estimation of forest height and canopy density from a single InSAR correlation coefficient. IEEE Geosci. Remote Sens. Lett..

[9] Leinss S., Bernhard P. (2021). TanDEM-X: Deriving InSAR height changes and velocity dynamics of great Aletsch Glacier. IEEE J. Sel. Top. Appl. Earth Obs. Remote Sens..

[10] Berthier E., Floriciou D., Gardner A.S., Gourmelen N., Jakob L., Paul F., Treichler D., Wouters B., Belart J.M.C., Dehecq A. (2023). Measuring glacier mass changes from space - A review. Rep. Prog. Phys..

[11] Huang L., Hajnsek I. (2024). A study of sea ice topography in the Weddell and Ross seas using dual-polarimetric TanDEM-X imagery. Cryosphere.

[12] Huang L., Fischer G., Hajnsek I. (2021). Antarctic snow-covered sea ice topography derivation from TanDEM-X using polarimetric SAR interferometry. Cryosphere.

[13] Gonzalez C., Rizzoli P., Milillo P., Dell’Amore L., Bueso-Bello J.L., Nagler T., Zink M. (2024). Proceedings European Conference on Synthetic Aperture Radar.

[14] Wessel B., Huber M., Wohlfart C., Bertram A., Osterkamp N., Marschalk U., Gruber A., Reuß F., Abdullahi S., Georg I., Roth A. (2021). TanDEM-X Polar DEM 90 m of Antarctica: Generation and error characterization. Cryosphere.

[15] Rodriguez-Cassola M., Prats P., Schulze D., Tous-Ramon N., Steinbrecher U., Marotti L., Nannini M., Younis M., Lopez-Dekker P., Zink M. (2012). First bistatic spaceborne SAR experiments with TanDEM-X. IEEE Geosci. Remote Sens. Lett..

[16] Prats P., López-Dekker P., De Zan F., Wollstadt S., Bachmann M., Steinbrecher U., Scheiber R., Reigber A., Krieger G. (2011). IEEE International Geoscience and Remote Sensing Symposium.

[17] Kraus T., Krieger G., Bachmann M., Moreira A. (2019). Spaceborne demonstration of distributed SAR imaging with TerraSAR-X and TanDEM-X. IEEE Geosci. Remote Sens. Lett..

[18] Kraus T., Bräutigam B., Bachmann M., Krieger G., Mittermayer J. (2016). Proceedings ONERA-DLR Aerospace Symposium.

[19] Richter D., Rodriguez-Cassola M., Zonno M., Prats-Iraola P. (2022). Proceedings 14th Eur. Conf. Synthetic Aperture Radar.

[20] Villano M., Peixoto M.N., Ustalli N., Mittermayer J., Krieger G., Moreira A. (2022). Decorrelating ambiguities in SAR interferometry through slight PRI variation. IEEE Trans. Geosci. Remote Sens..

[21] Baumgartner S.V., Krieger G. (2016). Dual-platform large along-track baseline GMTI. IEEE Trans. Geosci. Remote Sens..

[22] Nannini M., Martone M., Rizzoli P., Prats-Iraola P., Rodriguez-Cassola M., Reigber A., Moreira A. (2019). Coherence-based SAR tomography for spaceborne applications. Remote Sens. Environ..

[23] Mittermayer J., Wollstadt S., Prats P., López-Dekker P., Krieger G., Moreira A. (2013). Bidirectional SAR imaging mode. IEEE Trans. Geosci. Remote Sens..

[24] Caldarella N., Lopez-Dekker P., Prats-Iraola P., Nouguier F., Chapron B., Zonno M., Rodriguez-Cassola M. (2022). Retrieval of wind and total surface current vectors using experimental bidirectional along-track interferometric TanDEM-X data. IEEE Trans. Geosci. Remote Sens..

[25] Li T., Tang X., Li S., Zhou X., Zhang X., Xu Y. (2023). Classification of basic deformation products of L-band differential interferometric SAR satellite. Acta Geod. Cartogr. Sinica.

[26] Zhang X., Tang X., Li T., Zhao H., Zhang X., Li L. (2023). Proceedings SAR Big Data Era.

[27] Ji Y., Zhang X., Li T., Fan H., Xu Y., Li P., Tian Z. (2023). Mining deformation monitoring based on LuTan-1 monostatic and bistatic data. Remote Sens..

[28] Torres R., Lokas S., Moller H.L., Zink M., Simpson D.M. (2004). Proceedings IEEE International Geoscience and Remote Sensing Symposium.

[29] Moreira A., Krieger G., Hajnsek I., Papathanassiou K., Younis M., Lopez-Dekker P., Huber S., Villano M., Pardini M., Eineder M. (2015). Tandem-L: A Highly Innovative Bistatic SAR Mission for Global Observation of Dynamic Processes on the Earth's Surface. IEEE Geosci. Remote Sens. Mag..

[30] Huber S., De Almeida F.Q., Villano M., Younis M., Krieger G., Moreira A. (2018). Tandem-L: A Technical Perspective on Future Spaceborne SAR Sensors for Earth Observation. IEEE Trans. Geosci. Remote Sens..

[31] Fiedler H., Boerner E., Mittermayer J., Krieger G. (2005). Total zero Doppler steering-A new method for minimizing the Doppler centroid. IEEE Geosci. Remote Sens. Lett..

[32] Boerner E., Fiedler H., Krieger G., Mittermayer J. (2004). Proceedings of the IEEE International Geoscience and Remote Sensing Symposium.

[33] Fiedler H., Boerner E.,, Mittermayer J., Krieger G. (2009).

[34] Fiedler H., Fritz T., Kahle R. (2008). Proceedings of 2008 International Conference on Radar.

[35] Scharf D.P. (2012). Analytical yaw-pitch steering for side-looking SAR with numerical roll algorithm for incidence angle. IEEE Trans. Geosci. Remote Sens..

[36] Krieger G., Zonno M., Rodriguez-Cassola M., Lopez-Dekker P., Mittermayer J., Younis M., Huber S., Villano M., De Almeida F.Q., Prats-Iraola P. (2017). IEEE International Geoscience and Remote Sensing Symposium.

[37] Krieger G., Zonno M., Mittermayer J., Moreira A., Huber S., Rodriguez-Cassola M. (2018). Proceedings of 12th EUSAR conference.

[38] Zonno M., Rodriguez-Cassola M., Krieger G., Moreira A., Lopez-Dekker P. (2019).

[39] Zonno M., Krieger G., Rodriguez-Cassola M., Mittermayer J., Moreira A. (2018). Proceedings of 12th EUSAR conference.

[40] Mittermayer J., Krieger G., Bojarski M., Zonno M., Villano M., Moreira A. (2021). Proceedings of 14th EUSAR conference.

[41] Mittermayer J., Krieger G., Moreira A. (2020). Proceedings of IEEE Radar Conference.

[42] Quiroz A.E.N., Bartusch M., Stettner S., Moreira A., Zink M. (2022). Proceedings of the 2022 IEEE International Geoscience and Remote Sensing Symposium.

[43] Mittermayer J., Krieger G., Bojarski A., Zonno M., Villano M., Pinheiro M., Bachmann M., Buckreuss S., Moreira A. (2022). Mirror-SAR: an HRWS add-on for single-pass multi-baseline SAR interferometry. IEEE Trans. Geosci. Remote Sens..

[44] Mittermayer J., Krieger G., Wollstadt S. (2022). Numerical calculation of Doppler steering laws in bi- and multistatic SAR. IEEE Trans. Geosci. Remote Sens..

